# Structural and Functional Brain Changes in Children and Adolescents With Obesity

**DOI:** 10.1111/obr.70001

**Published:** 2025-08-04

**Authors:** Sixiu Zhao, Lorenzo Semeia, Ralf Veit, Julia Moser, Hubert Preissl, Stephanie Kullmann

**Affiliations:** ^1^ Institute for Diabetes Research and Metabolic Diseases Helmholtz Center Munich at the University of Tübingen Tübingen Germany; ^2^ German Center for Diabetes Research (DZD) Tübingen Germany; ^3^ fMEG‐Center Eberhard Karls University of Tübingen Tübingen Germany; ^4^ Masonic Institute for the Developing Brain University of Minnesota Minneapolis Minnesota USA; ^5^ Department of Internal Medicine, Division of Diabetology, Endocrinology and Nephrology Eberhard Karls University Tübingen Tübingen Germany; ^6^ Department of Pharmacy and Biochemistry Eberhard Karls University Tübingen Tübingen Germany

**Keywords:** adolescents, brain, children, magnetic resonance imaging, obesity

## Abstract

Obesity, particularly pediatric obesity, has dramatically increased over the last three decades, with a wide range of detrimental health outcomes, including negative consequences for brain neurodevelopment. The present article reviewed magnetic resonance imaging studies between January 2011 and March 2024 examining the brain's role in pediatric obesity, including parental influences and diverse interventions. A literature search identified 97 eligible MRI studies in the pediatric population. Findings suggest that altered brain structures and functions in pediatric obesity are strongly dependent on the developmental stage of children and adolescents. The function and structure of limbic regions, such as the hippocampus, amygdala, and striatum, as well as the prefrontal cortex, seem to be particularly affected by higher body mass index during development. In response to palatable foods, children and adolescents with excess weight have increased activation in reward‐related regions and decreased activation in regions involved in interoceptive signal processing, especially during decision processes. In addition, children of mothers with obesity and gestational diabetes mellitus show alterations in brain structure and function independent of their current obesity. Behavioral, exercise, and weight‐loss intervention studies showed promising effects on the brain, with increased structural integrity, decreased brain responses to reward, and strengthened inhibitory brain responses in children and adolescents with excess weight after the intervention.

**Abbreviations**
ASL: arterial spin labeling
BMI: body mass index
DTI: diffusion tensor imaging
FA: fractional anisotropy
FC: functional connectivity
fMRI: functional MRI
GDM: gestational diabetes mellitus
MeSH: Medical Subject Headings
MRI: magnetic resonance imaging
NAcc: nucleus accumbens
OFC: orbitofrontal cortex
PFC: prefrontal cortex
ROI: region of interest
rs‐fMRI: resting state fMRI
TS: topic
TW: text word

## 1 Introduction

The global prevalence of overweight (body mass index [BMI] percentile ≥ 85th) and obesity (BMI percentile ≥ 95th) among children and adolescents has risen dramatically, rising from 8% in 1990 to 20% in 2022 [1]. Pediatric obesity could persist into adulthood, and it increases the risk of developing type 2 diabetes, cardiovascular disease, and cancer later in life [2].

During this developmental phase, the brain undergoes rapid and dynamic development, characterized by heightened neuroplasticity [3]. Magnetic resonance imaging (MRI) enables the estimation of brain size, microstructure, and the function of specific systems throughout childhood and adolescence. It has been shown that the volume of cortical gray matter generally develops in an “inverted U” pattern, which increases during early childhood and declines in postadolescence, with the latest maturation in the prefrontal cortex (PFC) (for reviews, see [4, 5, 6]). Mesolimbic regions, such as the basal ganglia and hippocampus/amygdala, exhibit the same developmental pattern, with peaking times at 10 and 14 years, respectively [6, 7, 8]. Cortical thickness keeps decreasing, and the integrity of white matter keeps increasing throughout childhood and adolescence [4]. Generally, both structural and functional studies indicate an earlier maturation in the mesolimbic reward regions, while the prefrontal control system develops later [4, 5].

Several neurobehavioral hypotheses for the development of obesity have been proposed, even though they are not exclusively focused on pediatric obesity (for review see [9]). For example, the incentive sensitization hypothesis suggests that overconsumption of high‐calorie food leads to increased response to food rewards in reward (e.g., striatum, amygdala, hippocampus, orbitofrontal cortex [OFC], and insula) and attentional regions (e.g., occipital cortex, fusiform gyrus, frontal operculum, and anterior cingulate cortex) via food reward learning, subsequently promoting overeating. The reward surfeit hypothesis posits that a hyperactivity to high‐calorie food tastes in the reward system contributes to overeating and obesity. In addition, the inhibitory control deficit hypothesis proposes that lower activation in inhibitory control regions (e.g., ventral lateral PFC, dorsolateral PFC) in response to immediate food rewards results in overeating. Despite relying on different circuitries, all these hypotheses of obesity imply an imbalance of reward and cognitive control systems.

The aim of this review is to provide a detailed insight into neuroimaging research (i.e., using structural and functional magnetic resonance imaging [MRI and fMRI]) examining the neural alterations related to pediatric obesity. Moreover, we consider how maternal or paternal metabolic status influences obesity onset in the offspring. Lastly, we review available nonpharmacological intervention strategies and their effect on brain structure and function in pediatric obesity. We will also evaluate the evidence for the different hypotheses regarding obesity.

## 2 Materials and Methods

### 2.1 Search Strategy

We reviewed MRI research on excess weight‐related neuro‐alterations among children and adolescents. To identify relevant studies, we searched the online database PubMed and Web of Science to include articles published between January 2011 and March 2024. Search terms are given in the Supporting Information. We screened the identified studies to see if they matched the review topic by reading the title and abstract and examined the remaining articles in detail. The study selection process is illustrated in Figure S1.

### 2.2 Selection Criteria and Data Extraction

In the current review, inclusion criteria for studies were as follows: (1) empirical research, (2) child (1–10‐years old) and/or adolescent samples (10–19‐years old), (3) MRI methodology (e.g., fMRI) was used, and (4) study sample includes children or adolescents with excess weight (BMI ≥ 25 kg/m^2^; BMI percentile ≥ 85th). The following studies were excluded: (1) review articles, case studies, or meeting abstracts; (2) participants with type 2 diabetes, Prader–Willi syndrome, and psychiatric/neurological disorders; (3) studies not written in English.

Extracted data included information regarding sample characteristics (i.e., age, sample size, Tanner stage, weight status, gender, and exclusion criteria), MRI methods (including MRI modality, task description, and covariates), interventions used, and a summary of key findings from the extracted data.

### 2.3 Selected Publications

**FIGURE 1:**
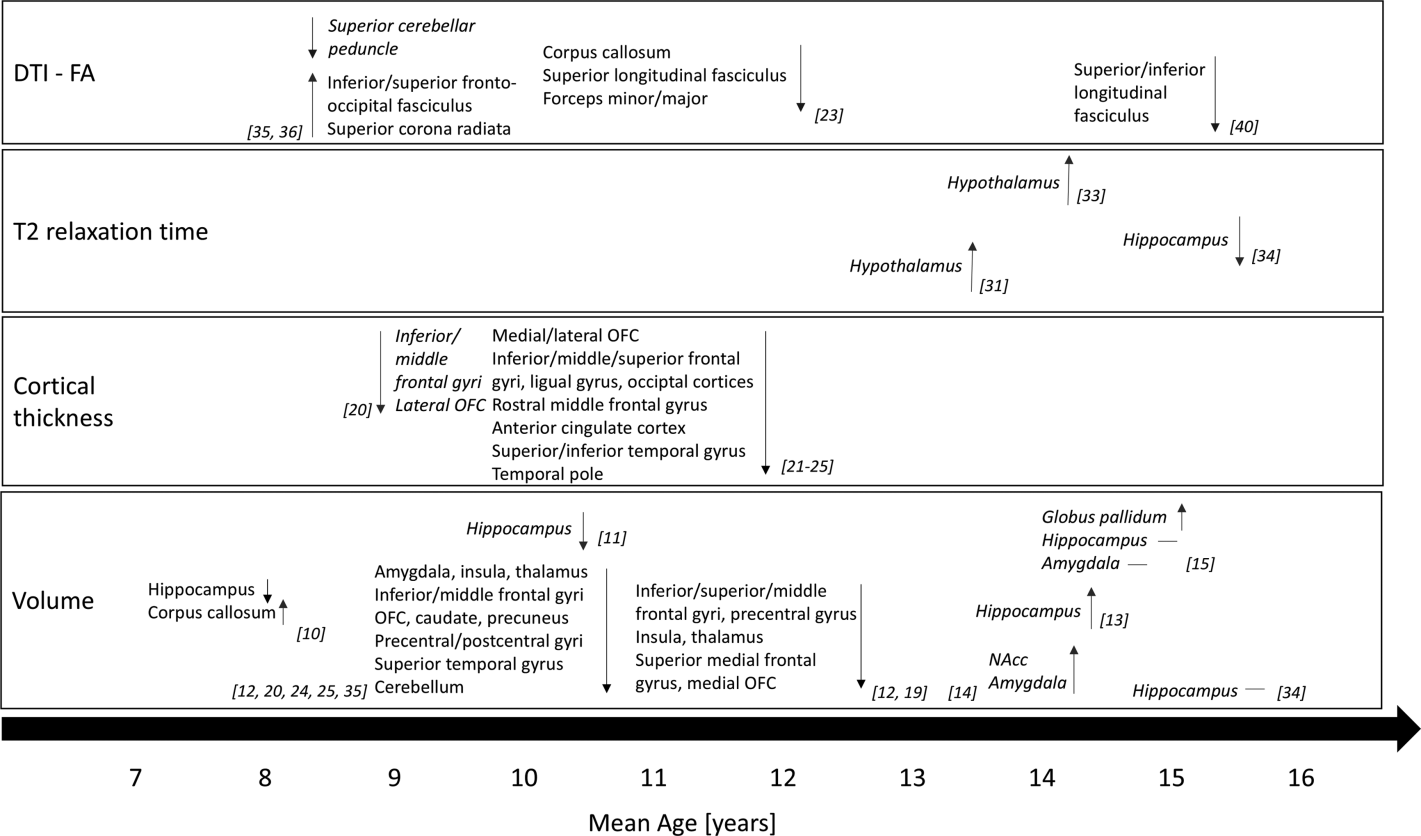
Structural alterations found in children and adolescents with excess weight compared to peers with healthy weight. DTI = diffusion tensor imaging; FA = fractional anisotropy; NAcc = nucleus accumbens; PFC = prefrontal cortex. “↑” means a greater metric. “↓” means a smaller metric. “—” means no changes in metrics. Straight font means that the study used whole‐brain analysis. Italic font means that the study used region of interest analysis.

**FIGURE 2:**
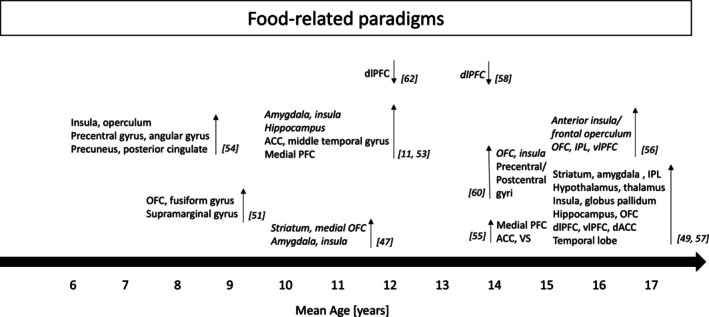
Functional alterations found during food‐related paradigms in children and adolescents with excess weight compared to peers with healthy weight. ACC = anterior cingulate cortex; dACC = dorsal anterior cingulate cortex; PFC = prefrontal cortex; dlPFC = dorsolateral prefrontal cortex; vlPFC = ventrolateral prefrontal cortex; OFC = orbitofrontal cortex; IPL = inferior parietal lobe; IFG = inferior frontal gyrus. “↑” means higher activation during different paradigms. “↓” means lower activation during different paradigms. Straight font means that the study used whole‐brain analysis. Italic font means that the study used region of interest analysis.

Thus far, 97 publications in total met the inclusion criteria. Most studies (*n* = 63) investigated the effect of obesity on brain structure and neural activity in children and adolescents (*n* = 23 assessed structural differences in both gray and white matter, *n* = 5 assessed microstructural differences in white matter, *n* = 1 detected both structural and white matter microstructural differences, *n* = 1 detected structural, white matter microstructural differences and intrinsic neural network using rs‐fMRI, *n* = 1 detected structural differences and intrinsic neural network, *n* = 25 examined neural activity during different paradigms, *n* = 1 examined both structure and neural activity, *n* = 6 examined intrinsic neural networks). In Tables 1 and 2, we summarize the characteristics of these studies. Figures 1 and 2 illustrate the major structural and functional brain alterations in children and adolescents with excess weight. Nine studies investigated the role of parental metabolic status on pediatric obesity. Twenty‐five studies examined the effects of physical fitness on brain health and different nonpharmacological interventions on the brains. The characteristics of these studies are summarized in Tables S1 and S2, respectively. Figure S2 describes the brain alterations in children and adolescents with excess weight after diverse interventions. We use the phrase excess weight for all participants above the normal weight range.

**TABLE 1:** Description of structural MRI and DTI studies in children and adolescents with excess weight.

Author	Study design	Study sample	Age (M, SD)/age range	Tanner stage (M)/range	Weight status: BMIz/BMI/BMI cole score/BMI% (M, SD)	Gender (% female)	Imaging modality	Metrics	Analysis methods	Main outcomes
Bauer et al. [10]	C.S.	33 (18 HW, 15 OW/OB)	All: 7.6 (0.42)	N.S.	BMI: HW: 15.29 (1.3); OW/OB: 21.61 (5.0)	64	Structural MRI	Volume	Whole‐brain analysis	Children with OW/OB vs. HW: ↓ **Hippocampus**, ↑ **corpus callosum**
Mestre et al. [11]	C.S.	25 (13 HW, 12 OB)	HW: 10.38 (1.26); OB: 10.08 (1.00)	N.S.	BMI: HW: 17.71 (1.9); OB: 26.10 (3.23)	40	Structural MRI	Volume	ROI analysis	Children with OB vs. HW: ↓ **Hippocampus**
Jiang et al. [12]	L.S.	523 (265 HW, 258 OB)	Month pre‐HW: 119.1 (0.4); OB: 119.5 (0.5); post‐HW: 142.8 (0.4); OB: 143.2 (0.5)	N.S.	BMI: pre‐HW: 16.8 (0.1); OB: 26.5 (0.2); post‐HW: 18.1 (0.1); OB: 30.0 (0.3)	48	Structural MRI	Gray matter volume	Whole‐brain analysis	Children with OB vs. HW (2‐year follow‐up vs. baseline): ↓ **Amygdala**, **caudate**, **OFC**, **thalamus, inferior parietal lobule**, **superior medial frontal gyrus**, **superior dorsolateral frontal gyrus**, **superior and middle frontal gyrus**, and **precentral gyrus** Children with OB vs. HW: ↓ **Amygdala**, **insula**, **superior temporal gyrus**, **middle cingulate cortex**, **inferior frontal gyrus**, **supplementary motor area**, **precuneus**, and **calcarine fissure**
Moreno‐Lo´pez et al. [13]	C.S.	52 (16 HW, 36 OW/OB)	HW: 14.13 (1.36); OW/OB: 14.22 (1.4)	N.S.	BMI: HW: 20.26 (2.8); OW: 24.85 (1.42); OB: 31.46 (2.91)	67	Structural MRI	Volume	ROI/Whole‐brain analysis	Adolescents with OW/OB vs. HW: **Hippocampus** ↑
Perlaki et al. [14]	C.S.	51 (in total)	All: 13.8 (1.9)	2–5	BMIz: all: 0.38 (1.24)	63	Structural MRI	Volume	ROI analysis	BMI z‐score **positively** correlated with the volume in the **NAcc** and **amygdala**
deGroot et al. [15]	C.S.	42 (19 HW, 23 OB)	12–16	N.S.	N.S.	N.S.	Structural MRI	Volume	ROI analysis	Adolescents with OB vs. HW: ↑ **Globus pallidum** No relationship between BMI and the volume of striatum, hippocampus, and amygdala
Hashimoto et al. [16]	L.S.	107 (in total)	Pre: 11.1; post: 14.1	N.S.	BMI: pre: 13.2–27.4; post: 12.5–37.1	47	Structural MRI	Volume	Whole‐brain analysis	During 3‐year development, BMI increase **negatively** correlated with increase of the volume in the **hippocampus** and **parahippocampal gyrus**
Migueles et al. [17]	C.S.	96 (in total)	All: 10.02 (1.13)	N.S.	BMIz: all: 3.04 (0.89)	40	Structural MRI	Volume	ROI/Whole‐brain analysis	Children with OW/OB: Total sleep time and sleep efficiency **positively** correlated with **hippocampal** volume; wake after sleep onset **negatively** correlated
Higgins et al. [18]	C.S.	149 (in total)	All: 8.99 (1.21)	N.S.	BMI%: All: 98.2 (17.5)	51	Structural MRI	Gray matter volume	ROI analysis	Exclusive breastfeeding duration **positively** correlated with **hippocampal** volume
Zhang et al. [19]	C.S.	476 (244 HW, 232 OW/OB)	HW: 144.34 (0.46); OW/OB: 144.16 (0.51)	N.S.	BMI: HW: 18.16 (0.11); OW/OB: 27.07 (0.29)	45	Structural MRI + working memory task	Gray matter volume	Whole‐brain analysis	Children with OW/OB vs. HW: ↓ Volumes in the **superior frontal gyrus**, **dorsal anterior cingulate cortex**, **medial superior frontal gyrus**, and **medial OFC** ↓ Activation in the **caudate**, **amygdala**, **insula**, and **superior temporal lobe** during working memory task
Hall et al. [20]	L.S.	11,226 (in total)	Month pre all: 119.1 (7.5); post all: 131.2 (7.7)	1–5	BMIz: pre all: 0.9 (2.2); post all: 1.4 (2.4)	48	Structural MRI	Gray matter volume/cortical thickness	ROI analysis	BMI z‐score **negatively** correlated with volume and thickness in the **inferior/middle frontal gyrus** and **lateral orbitofrontal cortex** at baseline Volume of **middle frontal gyrus** at baseline **negatively** correlated with BMI z‐score increase 1 year later
Laurent et al. [21]	C.S.	3190 (in total)	All: month 120.2 (7.3)	1–5	BMI: all: 18.64 (3.9)	49	Structural MRI	Cortical thickness	Whole‐brain analysis	BMI **negatively** correlated with cortical thickness in the **OFC**, **pars triangularis**, **frontal pole**, **rostral middle frontal gyrus**, **superior frontal gyrus**, **superior/inferior temporal gyrus**, and **temporal pole**
Ronan et al. [22]	C.S.	2700 (in total)	9–11	N.S.	N.S.	50	Structural MRI	Cortical thickness	Whole‐brain analysis	BMI **negatively** correlated with cortical thickness in the **OFC**, **inferior frontal gyrus**, **rostral middle frontal gyrus**, **superior frontal gyrus**, and **temporal pole**
Kaltenhauser et al. [23]	L.S.	4576 (in total)	All: month 119.8 (7.6)	1.5 (0.5)	BMIz: all: 0.3 (1.1)	48	Structural MRI + DTI + fMRI	Cortical thickness/FA/rs‐FC	Whole‐brain analysis	At baseline and second year: BMI and waist circumference **negatively** correlated with cortical thickness in the **middle frontal gyrus**; FA in the **corpus callosum**, **superior longitudinal fasciculus**, and **forceps minor/major**; FC in the **salience network** BMI at baseline **negatively** correlated with FA in the **inferior‐fronto‐occipital fasciculi**, **anterior thalamic radiations**, and **corpus callosum** in 2 years
Brooks et al. [24]	C.S.	4922 (in total)	All: month 120.0 (13.0)	1–4	BMIz: HW: −0.46 (0.57); OW: 0.66 (0.40); OB: 1.75 (0.97)	52	Structural MRI + fMRI	Cortical thickness/volume/rs‐FC	Whole‐brain analysis	Children with OW/OB vs. HW: ↓ Volume and thickness in the s**uperior/middle frontal gyrus**, **anterior cingulate cortex**, **medial/lateral OFC**, and **lateral occipital cortices**; ↓ FC, topological efficiency, connectedness, clustering in **default mode network**, **dorsal attention network**, **salience network**, and **executive control network**
Cui et al. [25]	C.S.	8484 (HW: 5759; OW: 1302 OB: 1423)	Month HW: 118.96 (0.10); OW: 119.14 (0.21) OB: 118.62 (0.20)	N.S.	BMI: HW: 16.73 (0.02); OW: 20.83 (0.03); OB: 26.13 (0.10)	58	Structural MRI	Cortical thickness/volume	Whole‐brain analysis	BMI **negatively** correlated with cortical thickness in the **lateral OFC**, **lingual gyrus**; cortical volume in the **pre/postcentral gyrus**, **precuneus**, **superior parietal lobule**, and **insula**
Gracia‐Marco et al. [26]	C.S.	100 (in total)	All: 10.0 (1.1)	N.S.	BMI: all: 26.7 (3.7)	40	Structural MRI	White/gray matter volume	Whole‐brain analysis	Children with OW/OB: Lean mass index **positively** correlated with white matter volume in the **putamen**, **superior frontal gyrus**, **superior fronto‐medial gyrus**, **middle fronto‐orbital gyrus**, **cerebellum**, and **parietal region**; gray matter volume in the **superior fronto‐orbital gyrus**
Sakib et al. [27]	L.S.	11,103 (in total)	All: 9.91 (0.6)	Pre: 1.7 (0.8) Post: 2.5 (1.0)	BMIz: pre all: 1.0 (2.4); post all: 1.9 (2.4)	48	Structural MRI	Gray matter volume/cortical thickness	ROI analysis	The volume and cortical thickness of **middle frontal gyrus** mediated the inverse association between executive function and BMI z‐score
Saute et al. [28]	C.S.	44 (26 HW, 18 OB)	HW: 16.81 (0.71); OB: 16.22 (0.73)	N.S.	BMI: HW: 21.38 (1.70); OB: 31.11 (3.21)	50	Structural MRI	Cortical thickness	Whole‐brain analysis	Visceral abdominal fat **positively** correlated with cortical thickness in the **superior frontal gyrus**, **superior temporal gyrus**, **pre/postcentral gyri**, **lateral occipital gyrus**, and **precuneus**
Adelantado‐Renau et al. [29]	C.S.	107 (in total)	All: 10.0 (1.1)	N.S.	BMI: all: 26.7 (3.7)	41	Structural MRI	Volume	Whole‐brain analysis	C‐reactive protein **negatively** correlated with **superior frontal gyrus** volume and **positively** correlated with **superior temporal gyrus** volume; Interleukin‐6 and tumor necrosis factor‐α **positively** correlated with **inferior frontal gyrus** volume; white blood cell **positively** correlated with **middle temporal gyrus** volume
Solis‐Urra et al. [30]	C.S.	96 (in total)	All: 10.0 (1.1)	N.S.	BMI: all: 26.7 (3.6)	38	Structural MRI	Gray matter volume	Whole‐brain analysis	Birth weight and birth length, as well as prolonged breastfeeding, **positively** correlated with increased volume in regions involved in higher‐order cognition and emotion regulation
Sewaybricker et al. [31]	C.S.	23 (12 HW, 11 OB)	HW: 13.3 (2.1); OB: 12.9 (2.7)	N.S.	BMIz: HW: 0.4 (0.8); OB: 2.1 (0.3)	48	Structural MRI	T2 relaxation time	ROI analysis	Children with OB vs. HW: **Hypothalamus** ↑
Sewaybricker et al. [32]	C.S.	31 (20 HW, 11 OW/OB)	All: 13.8 (2.5)	N.S.	BMIz: all: 0.75 (1.10)	61	Structural MRI	T2 relaxation time	ROI analysis	BMI z‐score **positively** correlated with T2 relaxation time in the **mediobasal hypothalamus**
Sewaybricker et al. [33]	L.S.	238 (114 OW, 124 OB)	OW: 9.9 (0.6) OB: 9.9 (0.6)	1–5	BMz: HW: 1.37 (0.17); OB: 2.04 (0.26)	47	Structural MRI	T2 relaxation time	ROI analysis	Children with OW, but not OB: **Hypothalamus/amygdala** T2 relaxation time **positively** correlated with adiposity gain over 1 year
Mestre et al. [34]	C.S.	102 (in total)	All: 15.07 (1.84)	N.S.	BMIz: All: 0.54 (1.17)	53	Structural MRI	T2 relaxation time + volume	ROI analysis	BMI z‐score **negatively** correlated with T2 signal intensity in the **hippocampus**; No relationship between BMI z‐score and hippocampal volume
Ou et al. [35]	C.S.	24 (12 HW, 12 OB)	HW: 9.8 (0.7); OB: 9.1 (0.9)	N.S.	BMI: HW: 15.8 (1.0); OB: 24.4 (3.4)	50	Structure + DTI	FA	Whole‐brain analysis	Children with OB vs. HW: ↑ FA in the posterior part of the **inferior** and **superior fronto‐occipital fasciculus** and **superior corona radiata** ↓ Volume in the **thalamus**, **middle temporal gyrus**, **pre/postcentral gyri**, **superior parietal gyrus**, and **cerebellum**
Augustijn et al. [36]	C.S.	44 (25 HW, 19 OB)	HW: 9.5 (1.2); OB: 9.4 (1.0)	N.S.	BMI: HW: 16.90 (1.15); OB: 31.03 (4.62)	34	DTI	FA	ROI analysis	Children with OB vs. HW: ↓ FA in the s**uperior cerebellar peduncle** and motor competence
Augustijn et al. [37]	L.S.	40 (22 HW, 18 OB)	Pre HW: 9.6 (1.2); OB: 9.5 (1.0); post HW: 10.0 (1.2); OB: 9.9 (1.0)	1–3	BMI: pre HW: 16.85 (1.15); OB: 31.64 (4.35); post HW: 16.93 (1.19); OB: 25.66 (3.68)	25	DTI	Network connected strength	Graph‐theoretical approach and network‐based statistics	Children with OB vs. HW: ↑ Structural connected strength between **putamen** and **caudate**, **NAcc** at baseline; No changes in the brain networks organization after multicomponent behavioral intervention
Solis‐Urra et al. [38]	C.S.	98 (in total)	All: 10.03 (1.16)	N.S.	BMI: all: 26.58 (3.64)	39	DTI	FA/MD	Whole‐brain analysis	Head circumference at birth **positively** correlated with FA in the **inferior fronto‐occipital fasciculus**, and **negatively** correlated with MD in the **cingulate gyrus part of cingulum**, **corticospinal**, **and superior thalamic radiation**
Li et al. [39]	C.S.	8842 (in total)	All: Month 119 (8)	1–4	BMI‐z: all: 0.4 (1.2)	49	DTI	RSI‐RND RSI‐RNI		Obesity mediates negative relationships between household socioeconomic status and maturation of the **inferior longitudinal fasciculus**, **anterior thalamic radiations**, and **forceps major**
Alarcón et al. [40]	C.S.	152 (88 HW, 64 OW/OB)	HW: 14.2 (0.1); OW: 13.8 (0.2) OB: 14.4 (0.4)	HW: 3.5 (0.1); OW: 3.5 (0.1); OB: 3.4 (0.2)	BMI%: HW: 58.9 (1.8); OW: 90.0 (0.4); OB: 96.9 (0.3)	43	DTI	FA	Whole‐brain analysis	BMI **positively** correlated with FA in the **superior/inferior longitudinal fasciculus**

*Note:* “↑” indicates a greater metric or an increase over time; “↓” indicates a smaller metric or a decrease over time.

Abbreviations: BMI = body mass index; BMIz = BMI z‐score; C.S. = cross‐sectional study; DTI = diffusion tensor imaging; FA = fractional anisotropy; fMRI = functional MRI; HW = healthy weight; L.S. = longitudinal study; M = mean; MD = mean diffusivity; MRI = magnetic resonance imaging; N.S. = not specified; NAcc = nucleus accumbens; OB = obesity; OFC = orbital frontal cortex; OW = overweight; RND = restricted normalized directional diffusion; RNI = restricted normalized isotropic diffusion; ROI = region of interest; rs‐fMRI = resting state fMRI; RSI = restriction spectrum imaging; SD = standard deviation.

**TABLE 2:** Description of fMRI studies in children and adolescents with excess weight.

Author	Study design	Sample size	Age (M, SD)/age range	Tanner stage (M)/range	Weight status: BMIz/BMI/BMI cole score/BMI % (M, SD)	Gender (% female)	Imaging modality	Paradigm	Fasting before fMRI	Analysis methods	Main outcomes
Borowitz et al. [41]	C.S.	164 (88 HW, 76 OW/OB)	HW: 14.14 (1.04); OW: 14.14 (1.01); OB: 14.58 (0.97)	N.S.	BMIz: HW: 0.14 (0.61); OW: 1.35 (0.17); OB: 2.06 (0.32)	53	fMRI	Resting state		ROI analysis	Adolescent obesity **positively** correlated with FC between the **medial OFC** and the **globus pallidum**, **olfactory tubercle**; **negatively** correlated with FC between the **medial OFC** and the **ventrolateral PFC**; and between the **hippocampus** and the **caudate**
Martín‐Pérez et al. [42]	C.S.	104 (51 HW, 53 OW/OB)	HW: 15.29 (1.75); OW/OB: 14.64 (1.78)	N.S.	BMI%: HW: 52.35 (24.35); OW/OB: 93.98 (3.98)	65	fMRI	Resting state		ROI analysis	Adolescents with OW/OB vs. HW: ↑ FC between the **hypothalamus** and the **OFC**, **VS**, **anterior insula**, and **middle temporal cortex**; ↓ FC between the **hypothalamus** and the **cerebellum**, **middle prefrontal**, and **precentral/postcentral gyri**
Moreno‐Lopez et al. [43]	C.S.	115 (55 HW, 60 OW/OB)	HW: 15.11 (1.82); OW/OB: 14.67 (1.70)	N.S.	BMI: HW: 20.84 (2.39); OW/OB: 29.26 (3.84)	61	fMRI	Resting state		ROI/whole‐brain analysis	Adolescents with OW/OB vs. HW: ↑ FC between the **middle temporal gyrus** and the **OFC**; ↓ FC between the **insula** and the **ACC**; ↓ FC between the **middle temporal gyrus** and the **PCC**
Black et al. [44]	C.S.	18 (9 HW, 9 OB)	HW: 12.3 (1.41); OB: 11.66 (0.87)	N.S.	BMI%: HW: 51.33 (20.43); OB: 97.80 (1.81)	56	fMRI	Resting state		ROI analysis	Children with OB vs. HW: ↑ FC between the **middle frontal gyrus** and the **ventromedial PFC** and the **lateral OFC**
Pujol et al. [45]	C.S.	230 (147 HW, 83 OW/OB)	All: 9.8 (0.9)	N.S.	BMI: all: 18.0 (2.8)	50	fMRI	Resting state		ROI analysis	Children with OW/OB vs. HW: ↓ FC between the **OFC** and the **NAcc**, **amygdala**
Solis‐Urra et al. [46]	C.S.	96 (in total)	All: 10.01 (1.14)	N.S.	BMI: all: 26.7 (3.69)	38	fMRI	Resting state		ROI analysis	Birth weight **positively** correlated with FC between the **hippocampus** and the **pre/postcentral gyri**, **cerebellum**; breastfeeding **positively** correlated with FC between the **hippocampus** and the **middle temporal gyrus**, and **negatively** correlated with FC between the **hippocampus** and the **primary motor cortex**, **angular gyrus**
Roth et al. [47]	C.S.	76 (22 HW, 54 OB)	HW: 10.4 (0.9); OB: 10.4 (0.8)	N.S.	BMI%: HW: 46 (18); OB: 98 (1.1)	45	fMRI	Visual food cue task	1st fMRI: eat 3 h prior fMRI;2nd fMRI: after a test meal	ROI/whole‐brain analysis	Children with OB vs. HW: ↑ Post‐meal food cue reactivity in the **medial OFC**, **VS**, **DS**, **amygdala**, **insula**, and **substantia nigra/VTA**, despite normal ghrelin responses
Adam et al. [48]	C.S.	12 (12 OW)	OW: 9.9 (1.1)	OW: 1–2	BMI: OW: 29.9 (5.7)	100	fMRI	Visual food cue task	Eat 3 h prior fMRI	Whole‐brain analysis	Girls with OW: Insulin sensitivity **negatively** correlated with food cue reactivity in the **ACC**, **OFC**, **insula**, **frontal operculum**, and **rolandic operculum**
Jastreboff et al. [49]	C.S.	40 (15 HW, 25 OB)	HW: 15.5 (1.38); OB: 15.69 (1.77)	HW: 4.6;OB: 4.2	BMI‐z: HW: 0.21 (0.46); OW/OB: 2.19 (0.34)	50	fMRI	Visual food cue task	Eat 2 h prior fMRI	Whole‐brain analysis	Adolescents with OB vs. HW: ↑ Food cue reactivity in the **striatal‐limbic regions**; All subjects: Leptin **positively** correlated with activation in the **motivation‐reward regions**
Yokum et al. [50]	C.S.	21 (in total)	All: 15.2 (1.18)	N.S.	BMI: all: 27.9 (5.16)	62	fMRI	Cognitive reappraisal strategies + visual food cue task	Eat 5 h prior fMRI	Whole‐brain analysis	Thinking of long‐term costs of eating the food, long‐term benefits of not eating the food, and suppressing food cravings during food cue task:↑activation in the **superior/middle frontal gyrus**, **ventrolateral prefrontal cortex**; ↓activation in the **posterior cingulate cortex**, **precuneus**
Masterson et al. [51]	C.S.	41 (25 HW, 16 OW/OB)	HW: 7.84 (0.68); OW/OB: 8.00 (0.73)	N.S.	BMI%: HW: 48.00 (18.00); OW/OB: 91.00 (5.00)	54	fMRI	Food cue task after food/toy commercial exposure	Eat 3 h prior fMRI	Whole‐brain analysis	Children with OW/OB vs. HW: ↑Food cue reactivity in the **OFC**, **fusiform gyrus**, and **supramarginal gyrus**
Jensen et al. [52]	C.S.	52 (29 HW, 23 OW/OB)	All: 15.96 (1.56)	N.S.	BMI%: HW: 54.55 (24.54); OW/OB: 93.78 (4.60)	N.S.	fMRI	Sleep restriction, food‐specific go/no‐go task	4 h	ROI/Whole‐brain analysis	Adolescents with NW, but not OW: Sleep restriction **positively** correlated with food cue reactivity in the **middle/inferior frontal gyrus**, **ACC**
Mestre et al. [11]	C.S.	25 (13 HW, 12 OB)	HW: 10.38 (1.26); OB: 10.08 (1.00)	N.S.	BMI: HW: 17.71 (1.9); OB: 26.10 (3.23)	40	fMRI	Tasting	Sated	ROI analysis	Children with OB vs. HW: ↑ Response to taste in the **hippocampus**
Boutelle et al. [53]	C.S.	23 (13 HW, 10 OB)	HW: 10.4 (0.3); OB: 9.9 (0.3)	N.S.	BMI%: HW: 53.9 (6.9); OB: 96.8 (0.5)	44	fMRI	Tasting	Sated	ROI/whole‐brain analysis	Children with OB vs. HW: ↑ Response to taste in the **amygdala**, **insula**, and **medial frontal cortex**
Bohon et al. [54]	C.S.	18 (10 HW, 8 OW)	6–8	N.S.	BMI%: HW: 5–73; OW: 88–95	72	fMRI	Tasting, visual food cue task	Eat 4–6 h prior MRI	Whole‐brain analysis	Children with OW vs. HW: ↑ Response to taste in the **insula**, **operculum**, **precentral gyrus**, **angular gyrus**, **precuneus**, and **posterior cingulate**; No differences for visual food cues
Yokum et al. [55]	C.S.	88 (10 HW, 8 OW)	HW: 14.6 (0.93); OW/OB: 14.5 (0.84)	N.S.	BMI: HW: 20.3 (2.0); OW/OB: 27.0 (2.8)	100	fMRI	Tasting	Eat 3–4 h prior MRI	Whole‐brain analysis	Adolescents with OW/OB vs. HW: ↑ Response to taste in the **medial frontal cortex**, **ventral anterior cingulate cortex**, and **VS**
Yokum et al. [56]	L.S.	39 (in total)	All: 15.6 (0.96)	N.S.	BMI: all: 24.2 (4.5)	100	fMRI	Food‐specific attention network task	Eat 4–6 h prior MRI	ROI analysis	BMI **positively** correlated with activation in the **anterior insula/frontal operculum** ↑, during reallocation of attention to appetizing food cues; Activation in the **OFC** at baseline predicted weight gain 1 year later
Moreno‐Padilla et al. [57]	C.S.	77 (39 HW, 38 OW/OB)	HW: 16.58 (1.63); OW/OB: 16.47 (1.66)	N.S.	BMI: HW: 21.36 (2.07); OW/OB: 29.89 (3.72)	52	fMRI	Food choice task	Eat 1–3 h prior MRI	Whole‐brain analysis	Adolescents with OW/OB vs. HW: ↑ Activation in the **striatum**, **OFC**, **globus pallidum**, **insula**, **hippocampus**, **dACC**, **dlPFC**, and **vlPFC** during choosing appetizing food (vs. plain food)
van Meer et al. [58]	L.S.	141 (in total)	All: 13.4 (1.8)	All: 2.43 (0.59)	BMI cole score: All 0.48 (1.04)	57	fMRI	Food choice task	Eat 2 h prior fMRI	ROI analysis	Age **positively**, but BMI **negatively** correlated with activation in the **dlPFC** during food choice Higher response in the attentional regions predicted greater weight gain per year
Lim et al. [59]	C.S	141 (in total)	All: 15.88 (0.93)	N.S.	BMI%: all: 71.98 (25.22)	0	fMRI	Food and physical activity decision task	4 h	Whole‐brain analysis	Adolescents with OW/OB vs. HW: ↓ Activations in the **inferior frontal cortex**, **motor cortex**, and **superior temporal gyrus** ↓ during physical activity decision making
Rapuano et al. [60]	C.S.	37 (19 HW, 18 OW/OB)	All: 14.4 (1.3)	N.S.	BMI: HW: 20.15 (2.05); OW/OB: 33.20 (2.51)	54	fMRI	Food/nonfood commercial	Eat 2 h prior fMRI	ROI/Whole‐brain analysis	Percent of body fat **positively** correlated with activation in the **OFC**, **insula**, and **mouth‐specific somatosensory‐motor cortices** to food commercial
Burger et al. [61]	C.S.	25 (in total)	All: 15.2 (0.8)	N.S.	BMI%: all: 67.4 (22.5)	48	fMRI	Food/nonfood commercial	4 h	Whole‐brain analysis	Soft drink commercial activated the **insula**, **putamen**, and **postcentral gyrus** in all children
Bruce et al. [62]	C.S.	20 (10 HW, 10 OB)	All: 11.85 (1.23)	N.S.	BMI%: HW: 50 (19.7); OB: 98.9 (1.7)	55	fMRI	Food/nonfood logo	Min. 4 h	Whole‐brain analysis	Children with OB vs. HW: ↓ Activation to food logos in the **middle/inferior prefrontal cortex**
Gearhardt et al. [63]	C.S.	171 (in total)	13–16	N.S.	N.S.	N.S.	fMRI	Fast food/nonfood commercial	N.S.	ROI/Whole‐brain analysis	Response to fast food commercials in the **NAcc**, **caudate**, and **hippocampus positively** correlated with subsequent food intake
Yokum et al. [64]	L.S.	40 (in total)	All: 15.2 (1.1)	N.S.	BMI: all: 26.9 (5.4)	57	fMRI	Food/nonfood commercial	5 h	ROI analysis	Activation to food commercial in the **striatum positively** correlated with BMI increases 1 year later
Navas et al. [65]	C.S.	68 (in total)	All: 16.56 (1.35)	N.S.	BMI%: all: 69.33 (28.83)	50	fMRI	Monetary incentive delay task	N.S.	ROI analysis	Adiposity **positively** correlated with response to general reward feedback in the **somatosensory regions**
Adise et al. [66]	C.S.	61 (30 HW, 31 OW/OB)	HW: 8.7 (1.4); OW/OB: 9.4 (1.2)	HW: 1.6 (0.8); OW/OB: 1.9 (1.1)	BMI%: HW: 53.4 (11.8); OW/OB: 94.5 (4.0)	54	fMRI	Modified card‐guessing task	Min. 3 h	ROI/Whole‐brain analysis	↑ **Striatal** response to winning money vs. food, regardless of weight status
Adise et al. [67]	C.S.	59 (31 HW, 28 OW/OB)	HW: 8.7 (1.4); OW/OB: 9.4 (1.2)	HW: 1.6 (0.8); OW/OB: 1.9 (1.1)	BMI%: HW: 53.4 (11.8); OW/OB: 94.5 (4.0)	54	fMRI	Modified card‐guessing task	Min. 3 h	Whole‐brain analysis	Brain response to food (vs. money) rewards in the **amygdala**, **OFC**, **medial PFC**, **positively** correlated with overeating, independent of weight status
Delgado‐Rico et al. [68]	C.S.	52 (16 HW, 36 OW/OB)	HW: 13.88 (1.36); OW: 14.07 (1.67); OB: 14.29 (1.31)	N.S.	BMI: HW: 20.19 (2.80); OW: 24.65 (1.26); OB: 31.33 (2.92)	50	fMRI	Risky‐gains task	N.S.	Whole‐brain analysis	Adolescents with OW/OB vs. HW: ↓ Activation in the **insula**, ↑ activation in the **midbrain**, during risky vs. safe choices; ↑ Activation in the **inferior frontal gyrus**, **parahippocampus**, **thalamus**, and **cerebellum**, during reward vs. punishment feedback
Mata et al. [69]	C.S.	54 (32 HW, 22 OW/OB)	HW: 15.53 (1.70); OW/OB: 15.14 (2.03)	N.S.	BMI: HW: 21.17 (2.24); OW/OB: 29.40 (3.00)	61	fMRI	Risky‐gains task	N.S.	ROI analysis	Adolescents with OW/OB: Activation in the **insula negatively** correlated with interoceptive sensitivity and restrained eating; activation in the **caudate positively** correlated with external eating
Verdejo‐García et al. [70]	C.S.	80 (44 HW, 36 OW/OB)	HW: 15.32 (1.69); OW/OB: 15.06 (1.88)	N.S.	BMI: HW: 20.96 (2.31); OW/OB: 29.11 (3.90)	61	fMRI	Social decision‐making task (ultimatum game)	N.S.	Whole‐brain analysis	Adolescents with OW/OB vs. HW: ↓ Activation in the **ACC**, **anterior insula**, **thalamus**, and **midbrain** during decisions about unfair (vs. fair) offers
Jensen et al. [71]	C.S.	42 (in total)	All: 16.48 (1.01)	N.S.	BMI%: all 94.57 (4.4)	100	fMRI	Sleep deprivation, Social stress induction task	4 h	ROI analysis	OW adolescent females with sleep deprivation: Sleep deprived **positively** correlated with activation in the **putamen**, **hippocampus** during social evaluation; Activation in the **ACC** during negative social feedback **positively** correlated with calorie consumption

*Note:* “↑” indicates a greater metric or an increase over time; “↓” indicates a smaller metric or a decrease over time.

Abbreviations: ACC = anterior cingulate cortex; BMI = body mass index; BMIz = BMI z‐score; C.S. = cross‐sectional study; dlPFC = dorsolateral prefrontal cortex; DS = dorsal striatum; FA = fractional anisotropy; FC = functional connectivity; fMRI = functional MRI; HW = healthy weight; L.S. = longitudinal study; M = mean; MRI = magnetic resonance imaging; N.S. = not specified; NAcc = nucleus accumbens; OB = obesity; OFC = orbitofrontal cortex; OW = overweight; PCC = posterior cingulate cortex; PFC = prefrontal cortex; ROI = region of interest; rs‐fMRI = resting state fMRI; SD = standard deviation; vlPFC = ventrolateral prefrontal cortex; VS = ventral striatum; VTA = ventral tegmental area.

## 3 Results

### 3.1 Evidences of Structural Differences in the Brain of Children With Excess Weight

Obesity impacts the brain structure in children. Previous studies measured different metrics, including volume, cortical thickness, and T2 relaxation time, to assess brain structural alterations in children with excess weight. Volumetric alterations in brain areas indicate changes in either the number or the size of cells in these areas [72]. Cortical thickness measures gray matter width, which is determined by synaptic density, as well as intracranial myelination [73]. In addition, T2‐weighted signal intensity can provide insights into changes in brain tissue properties by detecting variations in water content, where hyperintense signals reflect gliosis [31, 74].

Our review includes 26 studies addressing structural alterations associated with pediatric obesity. Volumetric alterations in mesolimbic regions, such as the hippocampus, amygdala, and basal ganglia (divided into globus pallidum and striatal divisions), have been linked to obesity, but results are inconsistent. For example, compared to their lean peers, children with excess weight showed lower volumes in the hippocampus and amygdala [10, 11, 12], while adolescents with excess weight (13–14 years) exhibited higher volumes in these regions [13, 14]. Similarly, a positive correlation between BMI and the volume of the globus pallidum and nucleus accumbens (NAcc, i.e., ventral striatum) was found in adolescents [14, 15]. However, other studies found no correlation between the volume of the amygdala, hippocampus, and obesity measures among older adolescents (14–16 years) [15, 34]. The observed inconsistency in mesolimbic regions between children and adolescents might arise from obesity disrupting the typical developmental pattern, characterized by an initial increase in the volume of these regions during early childhood, followed by a decline during adolescence [4]. However, longitudinal studies in both children and adolescents (spanning puberty) with excess weight showed a greater reduction in the volume of the amygdala, hippocampus, and caudate (dorsal striatum) after a 2‐year [12] and 3‐year [16] follow‐up, respectively, suggesting a potential detrimental impact of obesity on brain growth. Notably, studying a wide age range from childhood to adolescence could mask interaction effects that change throughout the developmental process. Interestingly, better sleep and longer breastfeeding duration were associated with larger hippocampal volume in children with excess weight [17, 18].

The structure of the PFC, a critical brain region related to executive function, has been widely studied in children and adolescents, employing large sample sizes. For instance, children with higher BMI or waist circumference exhibited decreased volume and cortical thickness in several regions of the PFC [19, 20, 21, 22, 23, 24, 25]. Lean mass index positively predicted PFC volume [26]. Furthermore, the reduced volume and thickness of the PFC partially mediated the inverse relationship between BMI and executive function (e.g., working memory) [21, 22, 27]. Additionally, children with excess weight showed a greater reduction of PFC volume and thickness over 2 years of development [12, 23]. Conversely, older adolescents exhibited a thicker PFC with increased visceral abdominal fat [28], implying a potential reversal in the developmental pattern of cortical thickness due to obesity. Based on consistent findings from the studies with large sample sizes [19, 20, 21, 22, 23, 24, 25], the observed association between obesity and structural alterations of the PFC suggests that this region is particularly vulnerable to the effects of pediatric obesity. The delayed maturity of the PFC compared to other brain regions during childhood and adolescence could be the reason [21]. However, given that the majority of studies were cross‐sectional in nature, a clear causal relationship between brain development and obesity remains elusive. In addition, inflammatory biomarkers [29] and adverse early life factors [30] were also related to decreased PFC volume in children with excess weight.

T2 relaxation time was used to investigate tissue properties, especially gliosis resulting from obesity, in the hypothalamus (homeostatic system) and hippocampus among adolescents. For example, adolescents with higher BMI exhibited longer T2 relaxation time in the hypothalamus [31, 32], suggesting the presence of hypothalamic gliosis, a response of the central nervous system to injury caused by obesity or high‐fat diet [31]. In addition, hypothalamic gliosis predicted weight gain over 1 year in adolescents with overweight but not with obesity, suggesting that gliosis could potentially precede the development of obesity [33]. On the contrary, a negative association between BMI z‐score and T2 relaxation time was found in the hippocampus [34]. Unlike gliosis, a decreased T2 relaxation time in the hippocampus might be due to the accumulation of macromolecules and lipids resulting from a high‐fat diet, resulting in heightened tissue viscosity [75]. However, there remains a research gap regarding the tissue properties in children with excess weight. Moreover, exploring additional brain regions, such as other limbic regions, could provide deeper insights into brain alterations related to pediatric obesity.

#### 3.1.1 Summary

**FIGURE 3:**
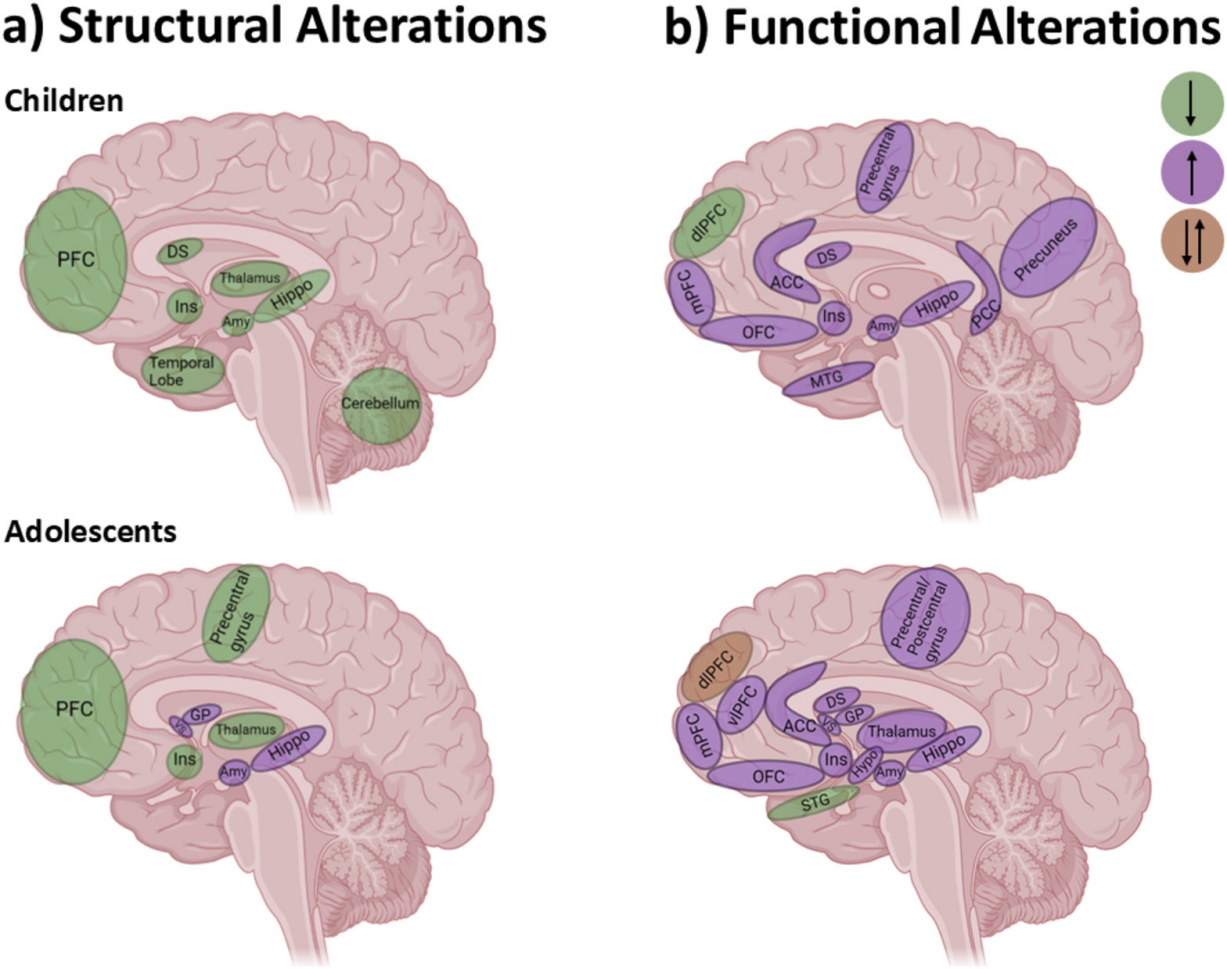
Altered brain structures and functions in pediatric obesity, compared to normal weight, are dependent on the developmental stage of the children and adolescents. (a) structural changes. (b) functional changes. ACC = anterior cingulate cortex; Amy = amygdala; DS = dorsal striatum; dlPFC = dorsolateral prefrontal cortex; GP = globus pallidum; Hippo = hippocampus; Hypo = hypothalamus; Ins = insula; MTG = middle temporal gyrus; OFC = orbitofrontal cortex; PFC = prefrontal cortex; PCC = posterior cingulate cortex; VS = ventral striatum; vlPFC = ventrolateral prefrontal cortex; STG = superior temporal gyrus.

According to these studies, children and adolescents with excess weight have altered volume, cortical thickness, or tissue properties mainly in the limbic regions, PFC, and hypothalamus. Although the results are mixed, multiple studies found decreased cortical thickness and volume in the PFC, accompanied by diminished executive function. Volumetric alterations in the hippocampus, amygdala, and basal ganglia are strongly dependent on developmental stage, with decreases observed in children and increases in adolescents (Figure 3a).

### 3.2 Evidences of White Matter Microstructural Differences in the Brain of Children With Excess Weight

Besides alterations in brain size, obesity‐related changes in white matter microstructure have also been investigated using diffusion tensor imaging (DTI), a neuroimaging technique that detects water diffusion at the cellular level [76]. Fractional anisotropy (FA) is the primary indicator of white matter integrity [76].

We identified seven studies linking excess weight to altered white matter integrity in children and adolescents. Association fibers, which connect the cortical regions within the same hemisphere, seem to be susceptible to obesity. For example, children with obesity aged 7–11 years showed higher FA in the association fibers, as well as lower FA in the superior cerebellar peduncle, compared to their lean peers [35, 36]. A structural network analysis revealed stronger connections within the ventral and dorsal striatum, as well as greater involvement of the precentral gyrus in this age group [37], suggesting that obesity impacts both the reward network and motor cortex. Although behavioral data on reward processing or motor skills were not examined, the graph theory‐based analysis provides additional insights into brain development in childhood obesity. Larger head circumference at birth and worse household socioeconomic status also influence the maturation of association fibers in these children [38, 39]. In older children and adolescents, however, a negative association was observed between BMI and FA in the association fibers and callosal fibers, with BMI negatively predicting FA in these fibers over a 2‐year development [23, 40]. Obesity may contribute to the inconsistency in FA values observed in the association fibers during development by disrupting the typical linear increase observed from childhood to adulthood. Additionally, FA values are determined by water diffusivity along three distinct axes, each of which can independently impact FA. Consequently, alterations in diffusion along these directions could also result in varied findings. However, given the limited research, especially in early adolescence, more studies are necessary to replicate the current findings.

### 3.3 Evidences of Functional Differences in the Brain of Children With Excess Weight

By assessing changes in the paramagnetic properties of hemoglobin, which depend on blood‐oxygen levels, fMRI, including task‐based fMRI and rs‐fMRI, can infer local neuronal activity and functional connectivity [77].

#### 3.3.1 Resting State fMRI

In comparison to task‐based fMRI, resting state functional connectivity (rs‐FC) provides insight into intrinsic neural networks [41].

There are a total of eight studies investigating rs‐FC in children and adolescents with excess weight. They showed altered rs‐FC between regions related to cognition and reward processing, with alterations in rs‐FC between reward‐related regions. These alterations are notably influenced by the developmental stage. For example, adolescents with excess weight showed decreased rs‐FC between cognitive regions (e.g., hippocampus and dorsolateral PFC) and reward regions (e.g., caudate and OFC) [41, 42, 43]; also increased FC among reward regions (e.g., pallidum and OFC) [41, 42]. While children with excess weight exhibited the opposite connectivity patterns [44, 45]. Reward regions are relatively more developed in adolescence than cognitive regions [78], and these reward areas might simply be underdeveloped in younger children, resulting in a lack of strong connections [41, 79]. However, these studies investigated connectivity patterns between specific regions based on researchers' hypotheses, rather than exploring the effects of obesity on brain‐wide connectivity. Only two studies using network analysis showed a negative association between BMI and rs‐FC within salience, executive control, and default mode networks in children with excess weight [23, 24]. Whether these brain alterations predict food craving behavior or subsequent weight gain was not examined. Early life factors, such as birth weight and breastfeeding, were also associated with hippocampal FC in children with excess weight [46].

In sum, the available literature suggests that adolescents with excess weight have weaker connectivity between regions involved in cognition and reward, and stronger connectivity between reward regions. Children with excess weight show an opposite pattern.

#### 3.3.2 Task‐Based fMRI

A variety of different tasks were used in relation to pediatric obesity. Specifically, food‐related paradigms including a visual food cue task (*n* = 5) [47, 48, 49, 50, 51], food‐specific go/no‐go task (*n* = 1) [52], tasting (*n* = 4) [11, 53, 54, 55], food‐specific attention network task (*n* = 1) [56], food choice task (*n* = 2) [57, 58], food and physical activity decision task (*n* = 1) [59], and a food commercial trigger (*n* = 5) [60, 61, 62, 63, 64] were used to examine eating behavior‐related brain responses in children and adolescents with excess weight. Furthermore, nonfood paradigms including a monetary incentive delay task (*n* = 1) [65], modified card‐guessing task (*n* = 2) [66, 67], risky‐gains task (*n* = 2) [68, 69], social decision‐making task (*n* = 1) [70], and a chatroom task (*n* = 1) [71] were used to investigate neural activity.

##### 3.3.2.1 Food Related Paradigms

Obesity is influenced by multiple factors, including genetics, health behaviors (e.g., physical activity), and socioeconomic status, with chronic overconsumption of calories also playing a role [1, 80]. Hence, brain responses to high‐calorie food cues are investigated to evaluate neural mechanisms of eating behavior. Children and adolescents with excess weight showed a higher response to visual high‐calorie food stimuli (vs. nonfood) in reward‐processing brain regions, including the OFC, ventral striatum, dorsal striatum, amygdala, substantia nigra/ventral tegmental area, and insula, compared to their lean counterparts [47, 49]. This effect was observed in both fasting [49] and sated states, despite an appropriate postprandial ghrelin response [47]. These findings align with the incentive sensitization hypothesis of obesity. The study design, which measures postprandial hormone levels and neural activation to a meal in a relatively large sample [47], contributes to understanding brain responses to postprandial hormones in childhood obesity. In addition, the responses in the reward regions were also positively correlated with leptin levels [49] and negatively correlated with insulin sensitivity [48], supporting the role of these hormones in regulating eating behavior. However, behavioral data such as food craving or food intake were not assessed in these studies. Additionally, different cognitive strategies, such as considering the benefits of not eating when confronted with high‐calorie food cues, resulted in increased activation in the inhibitory control regions and decreased activation in the attention‐related regions among adolescents, independent of their BMI [50]. Despite its small sample size, the study could provide a theoretical framework for developing effective interventions, such as cognitive training. Interestingly, sleep restriction increased food cue‐related reward processing independent of weight status during a food‐specific go/no‐go task [52].

In addition, elevated responses in regions related to reward, gustation, and attention to appetitive taste (vs. water) predicted increased eating in the absence of hunger in children with excess weight [11, 53, 54, 55], suggesting an increased sensitivity of gustation. These results support the reward surfeit hypothesis of obesity, highlighting the significance of brain responses to food taste in the development of obesity.

An attention bias to food in adolescents with excess weight was found as well [56]. Specifically, during a food‐specific attention task, adolescents with higher BMI not only exhibited a faster response to food stimuli but also a hyperactivity in the reward and attentional regions to appetitive food pictures. Moreover, these elevated responses predicted weight gain 1 year later [56]. These findings also support the incentive hypothesis of obesity, emphasizing the role of reward and attentional regions for weight gain. Notably, only female adolescents were included in this study.

Caloric intake is also controlled by decisions [68]. Adolescents with higher BMI exhibited decreased activation in the inhibitory control regions when choosing appetizing food [58] and physical activity images [59], which is consistent with the hypothesis of an inhibitory control deficit. Higher response in the attentional regions during food choice predicted greater weight gain per year [58], aligning more closely with the incentive sensitization hypothesis. However, increased response in the inhibitory control regions during food choice was found in older adolescents [57]. Again, the inconsistent results could arise from the interaction between obesity and developmental trajectory. For example, the older the adolescent, the more mature the inhibitory control regions, potentially prompting more deliberate attempts to suppress appetite, resulting in a higher reaction to food rewards [57]. Alternatively, studies with smaller sample sizes could contribute to the replication crisis, serving as another potential reason.

Brain responses to food commercials (e.g., food advertisements or logos), identified as significant contributors to children's eating behavior leading to obesity [81], were also examined, even though they are not standardized paradigms. Greater response to food commercials in reward and attentional regions predicted greater subsequent high‐calorie food intake and weight gain 1 year later in children and adolescents [60, 61, 63, 64]. The effects of excess weight, however, were not specifically investigated in these studies. Other studies showed that children with excess weight had increased activation in attentional regions and decreased activation in inhibitory control regions compared to their lean peers [51, 62]. These results are consistent with both incentive sensitization and inhibitory control deficit hypotheses of obesity. It appears that the rewarding properties of food commercials affect all children, but the impact is more pronounced in those with excess weight, potentially diminishing their self‐control. Reducing exposure to food commercials in their daily life could be an effective intervention.

###### 3.3.2.1.1 Summary

Taken together, children and adolescents with excess weight show hyperactivity in regions related to reward, gustation, and attention to external food cues and taste. These responses are predictive for subsequent overeating and weight gain. We therefore suggest that current findings are more consistent with the incentive sensitization hypothesis of obesity, emphasizing the role of reward regions in the development of pediatric obesity. The findings regarding activation in inhibitory control regions were mixed (Figure 3b).

##### 3.3.2.2 Nonfood Paradigms

To examine whether the association between pediatric obesity and hyperactivity in reward‐related regions is specific to food or general to all rewards, studies employed paradigms involving nonfood (e.g., monetary) rewards. Adolescents with higher body fat had decreased activation in the somatosensory cortex to monetary reward feedback [65]. However, no association was observed between weight status and the brain's response to either monetary or food rewards in children [66, 67]. Given that children's brains exhibit less maturity compared to those of adolescents, it is possible that the effects of weight status on neural processing to food vs. monetary reward were not fully distinguished in children [66]. Different task designs in children versus adolescents could be another possible reason for the inconsistent results.

Maximizing reward at the cost of risk seems to characterize the adolescent brain [82]. Among adolescents with excess weight, decreased activation in the risk‐signaling region (i.e., insula) to risky choices predicted reduced interoceptive sensitivity and increased external eating. Increased activation in the reward region compared to their lean peers was observed as well [68, 69]. These findings may suggest a higher focus on the reward properties than on the possible long‐term risks associated with a decision in adolescents with excess weight, resulting in a propensity to choose high‐calorie foods in their daily life.

In addition, neural responses in adolescents with excess weight have been found to be influenced by social factors, stress, and sleep. Obesity in adolescents decreased brain responses to unfair monetary offers in reward and emotional regions during a social decision‐making task, suggesting reduced emotional monitoring of social unfairness [70]. Social stress was also related to obesity. For instance, among girls with excess weight who were sleep deprived, both positive and negative peer evaluations were linked to increased brain activity in emotion‐related regions. This was interpreted as them paying more attention to social feedback [71].

In sum, no relationship was observed between obesity and brain responses to other forms of reward in children, but a negative correlation was identified in adolescents. Decreased activation in interoceptive signal processing regions and increased activation in reward regions during decision‐making were also found in adolescents with excess weight. However, caution is warranted in interpreting these results due to the limited number of studies and the variability in task designs.

### 3.4 The Role of Parents on Brain's Function and Structure of Children

Evidence indicates that genetics, epigenetics, shared environment, and other factors contribute to weight gain [83]. Parental obesity is a reliable predictor of offspring obesity risk in childhood, adolescence, and adulthood [84]. Exposure to maternal gestational diabetes mellitus (GDM) and obesity in utero also increases risk for obesity in offspring [85]. Our review provided nine studies addressing the role of mothers on the brain response of children and adolescents at high risk for excess weight.

#### 3.4.1 Parental Obesity and GDM

Even lean adolescents with obese mothers (high‐risk) showed a reduced volume or cortical thickness in the somatosensory and taste cortex, as well as inhibitory control regions, compared to lean peers of normal‐weight mothers (low‐risk) and peers with obesity [86]. Independent of their current adiposity, children also showed weaker activation in inhibitory control regions in response to food cues, suggesting weakness in inhibitory control circuitry could play a role in the intergenerational effects of obesity [87, 88]. Although these studies examined different brain parameters (volume/cortical thickness vs. food cue reactivity), both consistently highlight changes in key brain regions involved in inhibitory control [86, 87]. Their rigorous design, which includes high‐ and low‐risk lean groups, as well as an obese group [86, 87], enables isolating the specific effects of maternal obesity exposure on children's brain development. Follow‐up studies with a larger sample size on high‐risk children are expected to further elucidate causal factors in the development of childhood obesity. Only one study thus far has investigated paternal influence on children's brains, and no relationship was found between children's food cue reactivity and paternal BMI [88].

Risk for developing obesity also derives from exposure to maternal metabolic disorders in utero. For example, the volume or radial thickness of the hippocampus [89, 90], middle frontal gyrus, and superior temporal gyrus [91] in children exposed to maternal prepregnancy obesity and GDM was decreased independent of their current BMI. Similarly, independent of children's adiposity, GDM exposure increased hypothalamic cerebral blood flow [92], and gliosis [93], as well as OFC activation in offspring [94], suggesting that both homeostatic and mesolimbic areas might be affected by GDM exposure. However, these studies are based on specific regions of interest. Future studies based on other hypotheses, such as dysfunction of the inhibitory control system in children exposed to GDM, are necessary.

Collectively, the included studies suggest that independent of their current adiposity, children of mothers with excess weight have a hypoactive inhibitory control circuitry, while those who are exposed to early GDM show hyperactivity in the homeostatic and reward system. Moreover, maternal metabolic disorders may induce hippocampal structural alteration in children. However, we are unable to fully depict the impact of parental metabolic status on children's brains, as studies examining children with normal weight but obese parents were not included in this review.

### 3.5 Nonpharmacological Interventions Against Pediatric Obesity

Several interventions have been developed to prevent and treat pediatric obesity. These interventions target physical activity, eating behavioral adaptations, or a combination of these. Our research provided 12 studies investigating the relationship between physical fitness and brain function/structure in children and adolescents with excess weight, and 13 other longitudinal studies addressing the effects of different interventions on their brain, including exercise.

#### 3.5.1 Physical Fitness and Exercise Intervention

In children and adolescents with excess weight, physical fitness—assessed through physiological parameters (i.e., cardiorespiratory/speed‐agility/muscular fitness) [95, 96, 97, 98, 99, 100, 101, 102, 103] and questionnaires [104, 105, 106]—was positively related to brain volume [95, 96, 102, 103], radial thickness [97], cortical thickness [98], global FA [101, 104, 106], and node clustering in the default mode, executive, and salience networks [105]. It was also linked to functional connectivity between regions in the default mode, ventral attention, and frontoparietal networks [99, 100]. These brain changes, in turn, were positively related to cognitive function (e.g., intelligence and academic performance) [95, 96, 97, 98, 99, 103]. Despite variability in sample size (ranging from 99 to 5955), these studies consistently demonstrated the benefits of physical activity on brain development, supported by accurate physiological parameters and brain measurements. Hence, exercise interventions are widely used to improve brain health in children and adolescents with excess weight.

In a relatively large sample of children with excess weight, a 20‐week aerobic and resistance exercise intervention had no effect on brain volume, despite a significant improvement in cognitive ability [107]. This may result from insufficient intervention time or alterations restricted to the cellular or molecular level [107]. Interestingly, the effects of exercise on the white matter microstructure were found. For example, compared to the sedentary group, an 8‐month aerobic exercise intervention increased FA in the frontotemporal and frontoparietal fiber tracts of children from baseline, a change that was also related to higher attendance in the exercise [108, 109]. In the same exercise program, reduced synchrony in the default mode network, executive control network, and motor network; altered brain activation in cognitive processing regions during an antisaccade/flanker task; and increased cognitive performance were also found after the intervention [110, 111]. This may suggest an improvement in brain specialization and efficiency with exercise [110, 111]. Similar results were shown in another 3‐month aerobic exercise intervention study [112]. Whether these children lost weight was not reported in the above studies.

#### 3.5.2 Eating Behavioral Strategies

The adaptation of eating behavior has been shown to be an effective strategy in managing weight [113]. For example, compared to breakfast‐skipping days, adolescents' brain responses to food (vs. nonfood) cues in reward/motivation regions were decreased following a 6 days of breakfast consumption. The brain activation in these regions was also positively related to appetite [114]. Another study showed a reduced food cue reactivity in reward and visual attention regions following glucose consumption after a 6 months of food intake reduction device training, which reduces portion size and eating speed by a feedback technique [115]. Even though findings from these studies may suggest a reduction of subsequent food intake, this parameter was not investigated in these studies, nor was weight loss. Notably, the reduced sensitivity to food rewards observed postintervention supports the incentive sensitization hypothesis of obesity, which highlights hypersensitivity in reward regions promoting overeating and obesity. A decrease in sensitivity to food rewards could suggest a normalization of the reward system, similar to that observed in children with normal weight.

#### 3.5.3 Weight‐Loss Program Intervention

Weight loss interventions, combining exercise with dietary restriction, cognitive behavioral therapy, family management, etc., have been used commonly to manage weight and reverse the effects of obesity on the brain. For example, after a 5‐month combined intervention including exercise, dietary restriction, and cognitive behavioral therapy, there was not only a significant weight loss, but cerebellar cortex and total gray matter volume were increased among children [116]. The study design, which included a control group of children with healthy weight not enrolled in the intervention, added value by allowing an assessment of whether the observed brain volume changes resulted from the program. In addition, adolescents who exhibited a greater increase in insula activation during a risky‐gain task from baseline to post a 12‐week similar intervention lost more weight. This implies that during risky decision‐making, the insula displayed heightened responsiveness in individuals who achieved greater weight loss, potentially suggesting a normalization of the interoceptive system [117]. Similarly, adolescents with greater reductions in BMI showed a normalization of the reward system after exercise and dietary intervention as well [118]. Moreover, decreased activation after a meal and lower rs‐FC in the appetitive regions before intervention predicted greater weight loss after 6 months [119] and 3 months [120], respectively. This may suggest a predictive role of the reward system for successful weight loss.

In summary, exercise is the most commonly used intervention form to improve brain health in children and adolescents with excess weight with widespread effects on brain structure and function. Eating behavioral adaptations result in decreased brain reward responses. A low response to reward and high response to risky decision making seem to predict the success of weight loss.

## 4 Discussion

Pediatric obesity is a concerning public health burden that needs to be addressed. MRI studies in children and adolescents with excess weight provide evidence of obesity‐related alterations in brain function and structure. Findings suggest that altered brain structure and function in pediatric obesity are strongly dependent on the age or developmental stage of the children and adolescents studied (Figure 3). Specifically, limbic regions, such as the hippocampus, amygdala, and striatum, exhibit decreased volume in children with excess weight, contrasting with increased volume in adolescents. Furthermore, studies consistently report decreased volume and cortical thickness in the PFC. In response to palatable foods, children and adolescents with excess weight have increased activation in reward and attention‐related regions and decreased activation in regions involved in interoceptive signal processing, especially during decision‐making processes. Activation patterns in inhibitory control regions vary inconsistently across studies. We suggest that these findings support the incentive sensitization hypothesis, given the hyperactivity of primarily reward‐related regions and the predictive role of these regions in future weight gain. In addition, children of mothers with obesity and GDM show similar alterations in brain structure and function independent of their current adiposity. Furthermore, exercise, eating behavioral adaptation, and weight‐loss intervention studies showed promising effects with increased structural integrity and decreased brain reward responses in children and adolescents with obesity after the intervention. However, postintervention weight loss in studies of exercise and eating behavioral adaptation was not reported. In weight loss programs, a decreased brain response to reward and an interoceptive system sensitive to risky decision‐making predict the success of weight loss. The findings summarized in this review can provide a theoretical framework for developing more effective interventions.

Obesity has a strong genetic basis, encompassing genes linked to early‐onset monogenic obesity in pediatric populations and polygenic obesity, many of which are expressed in the brain [121]. These obesity‐related genes are thought to influence body weight primarily through centrally mediated effects [121]. A recent review highlighted two distinct neural pathways in the brain's regulation of energy balance in monogenic and polygenic forms of obesity, based on gene expression in specific brain regions [121]. For monogenic obesity, such as leptin deficiency and Prader–Willi syndrome, an impaired hypothalamic pathway involved in appetite control has been identified [121]. However, neuroimaging studies in children suggest that structural and functional alterations extend beyond the hypothalamus, impacting other areas, including the mesolimbic circuitry [122, 123, 124]. In contrast, polygenic obesity is thought to involve disruption in the reward processing pathway [121], with many obesity‐associated genes being highly expressed in the mesolimbic circuitry [125]. This aligns with the incentive sensitization hypothesis and is supported by MRI studies in pediatric populations, which reveal reduced brain volume in regions within this circuitry [126, 127].

The review indicates neural changes in children and adolescents with excess weight; nevertheless, several limitations persist. There is a considerable variation in sample sizes, such as from 12 [48] to 230 [45] among fMRI studies. It is crucial to question whether findings from these smaller studies accurately represent the effects of population‐level alterations. Power analyses should be conducted to ensure appropriate sample sizes for research. In addition, variations in fasting durations before task‐based fMRI measurement (from satiety to 6 h) and exclusive focus on specific sex [48, 56, 59] require caution while interpreting certain findings. Moreover, reward‐related tasks were typically performed in children, whereas adolescents were engaged in tasks related to attention and decision‐making. This could also lead to skewed findings. Furthermore, limited longitudinal studies prevent us from reaching a conclusion regarding long‐term brain outcomes resulting from disruption by obesity. Finally, despite evidence showing the relationship between pubertal measures/hormones and brain structuring during adolescence [128], no studies have investigated the specific impacts of pubertal status on the brains of children and adolescents with excess weight.

Future research may benefit from task designs that focus more specifically on inhibitory control, to examine how self‐regulation is affected by appetizing food cues. Furthermore, the connection between pediatric obesity and sex remains uncertain, highlighting the need for investigating potential sex‐based distinctions in future research. Finally, it will also be necessary to conduct more longitudinal neuroimaging studies in order to determine whether the observed alterations are a cause or consequence of the excess weight. The impact of obesity on the developmental trajectory of the brain in children and adolescents should also be investigated.

## Conflicts of Interest

The authors declare no conflicts of interest.

## Supporting information


**Figure S1:** Flow chart of study selection process.
**Table S1:** Description of studies investigating the role of parental metabolic status on pediatric obesity.
**Table S2:** Description of studies examining effects of different nonpharmacological interventions on the brains of children and adolescents with excess weight.
