# Supplementary material for: Structural and Functional Brain Changes in Children and Adolescents With Obesity
Source: Obes Rev. 2025 Aug 4;26(12):e70001. doi: 10.1111/obr.70001 (PMC12620109; doi:10.1111/obr.70001)
Supplement: Supplementary file 1 — Figure S1: Flow chart of study selection process. Table S1: Description of studies investigating the role of parental metabolic status on pediatric obesity. Table S2: Description of studies examining effects of different nonpharmacological interventions on the brains of children and adolescents with excess weight. [file OBR-26-e70001-s001.pdf]

Structural and functional brain changes in children and adolescents with obesity

Sixiu Zhao[1,2,3], Lorenzo Semeia[1,2,3], Ralf Veit[1,2,3], Julia Moser[1,2,4], Hubert Preissl[1,2,3,5,6], Stephanie Kullmann[1,2,3,5]

[1]Institute for Diabetes Research and Metabolic Diseases of the Helmholtz Center Munich at the University of Tübingen, Tübingen, Germany

[2]German Center for Diabetes Research (DZD), Tübingen, Germany

[3]fMEG-Center, Eberhard Karls University of Tübingen, Tübingen, Germany

[4]Masonic Institute for the Developing Brain, University of Minnesota, Minneapolis, MN, USA

[5]Department of Internal Medicine, Division of Diabetology, Endocrinology and Nephrology, Eberhard Karls University Tübingen, Tübingen, Germany

[6]Department of Pharmacy and Biochemistry, Eberhard Karls University Tübingen, Tübingen, Germany

Correspondence: Stephanie Kullmann, Institute for Diabetes Research and Metabolic Diseases of the Helmholtz Center Munich at the University of Tübingen, Otfried-Müller-Str. 47, 72076 Tübingen, Germany; Email: Stephanie.Kullmann@med.uni-tuebingen.de; Phone: 0049 7071 2987703

Supplementary Text

Search terms for PubMed were as follows: ((neuroimaging [MeSH]) OR (magnetic resonance imaging [MeSH]) OR (diffusion tensor imaging [MeSH]) OR (neuroimaging [TW]) OR (magnetic resonance imaging [TW]) OR (fMRI [TW]) OR (functional MRI [TW]) OR (functional magnetic resonance imaging [TW]) OR (resting state [TW]) OR (functional connectivity [TW]) OR (structure [TW]) OR (structural [TW]) OR (volume [TW]) OR (diffusion tensor imaging [TW]) OR (DTI [TW]) OR (Arterial Spin Labeling [TW]) OR (ASL [TW])) AND ((child [MeSH]) OR (adolescent [MeSH]) OR (child [TW]) OR (children [TW]) OR (adolescent [TW])) AND ((obesity [MeSH]) OR (obesity [TW]) OR (obese [TW])). Search terms for Web of science were as follows: (((((((TS=(neuroimaging)) OR TS=(magnetic resonance imaging)) OR TS=(fMRI)) OR TS=(resting state)) OR TS=(functional connectivity)) OR TS=(structure)) OR TS=(volume)) AND ((((TS=(children and obesity)) OR TS=(adolescent and obesity)) OR TS=(children and obese)) OR TS=(adolescent and obese)). Additional potential studies were searched in the reference sections of eligible articles.

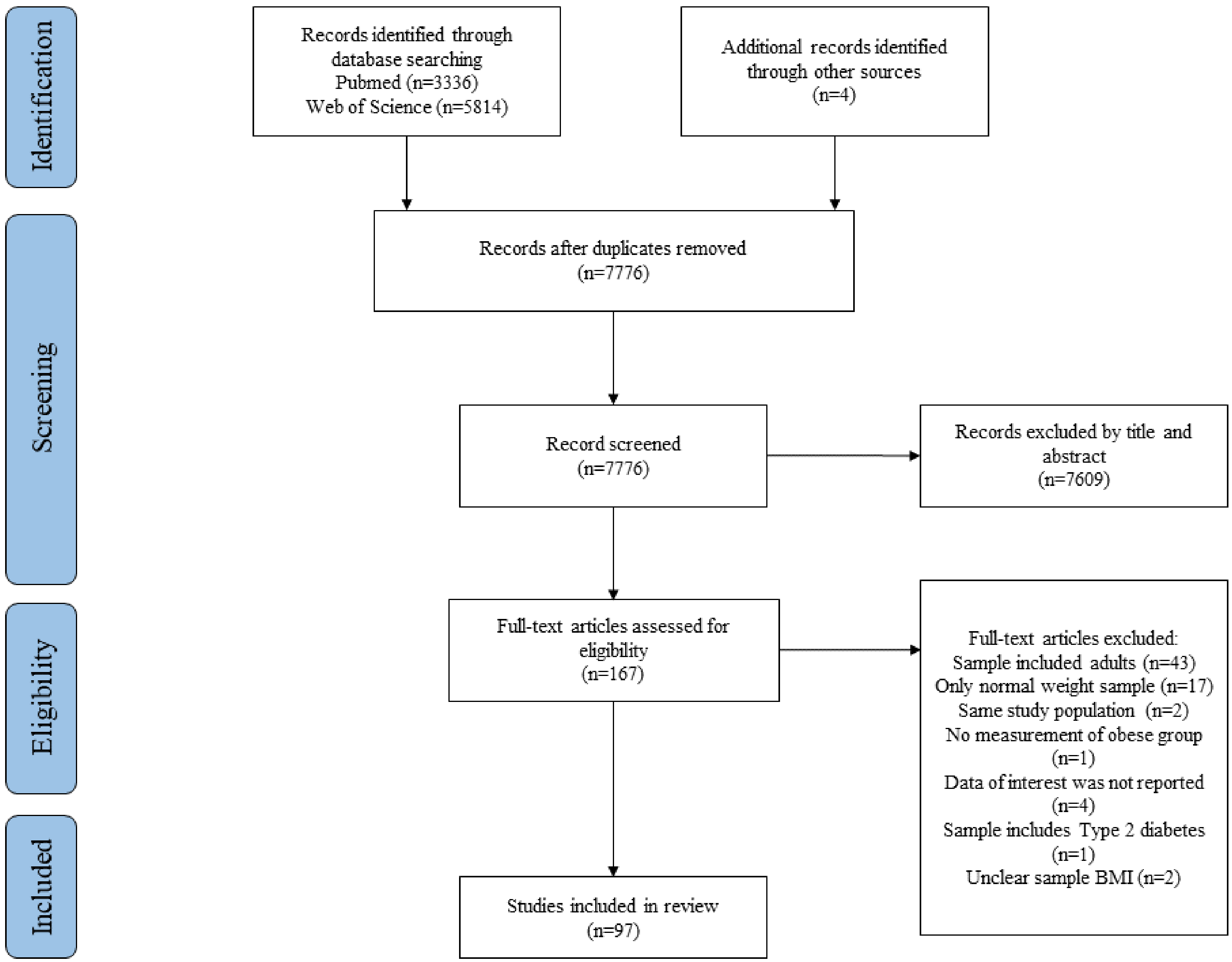

Supplementary Figure 1

Flow chart of study selection process.

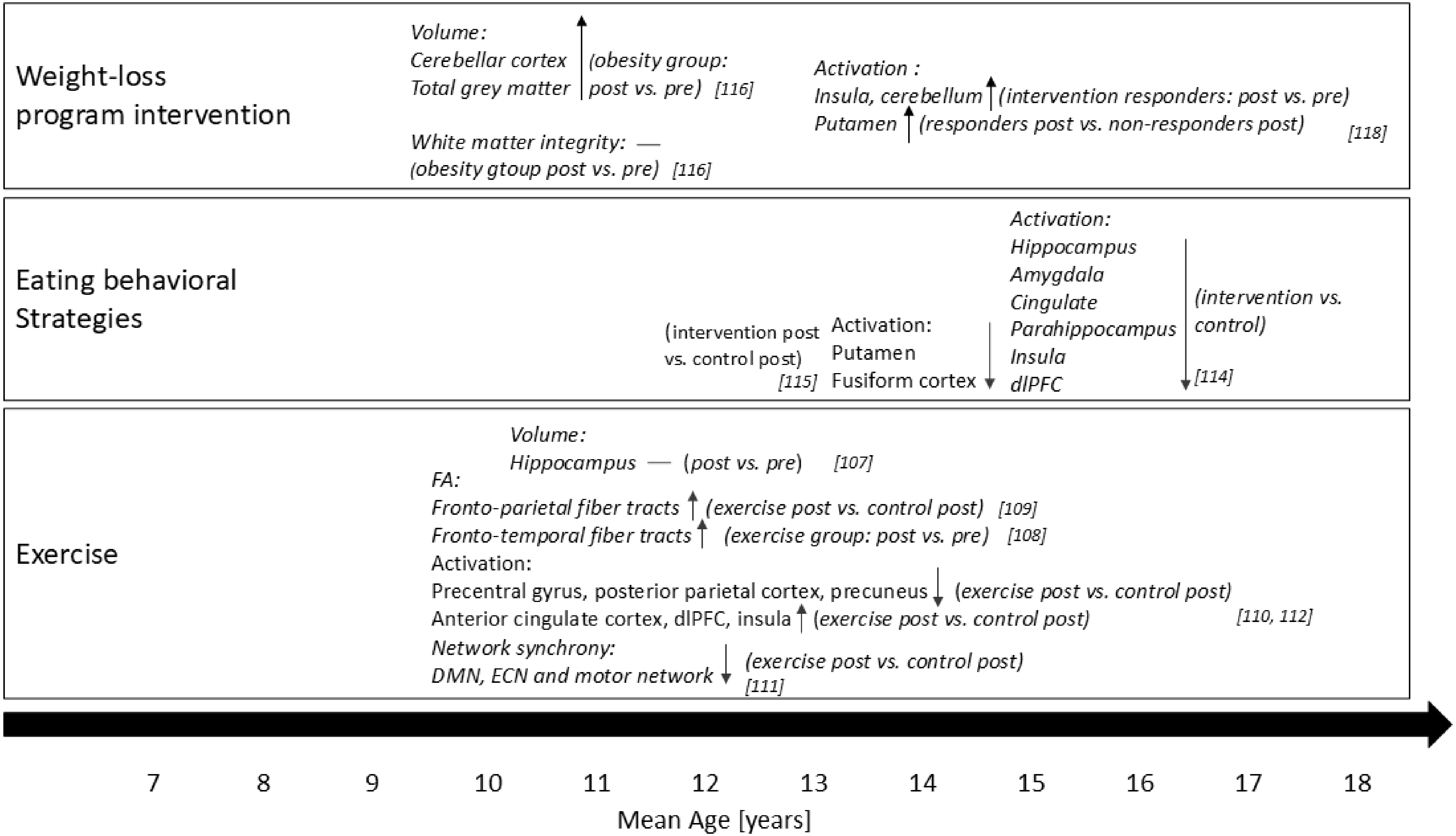
Weight-loss program intervention
Volume:
Cerebellar cortex
Total grey matter
(obesity group: post vs. pre) [116]
White matter integrity: —
(obesity gtoup post vs. pre) [116]
Activation :
Insula, cerebellum (intervention responders: post vs. pre)
Putamen (responders post vs. non-responders post)
[118]
Eating behavioral Strategies
(intervention post vs. control post)
[115]
Activation:
Putamen
Fusiform cortex
Activation:
Hippocampus
Amygdala
Cingulate
Parahippocampus
Insula
dlPFC
(intervention vs. control)
[114]
Exercise
Volume:
Hippocampus — (post vs. pre) [107]
FA:
Fronto-parietal fiber tracts (exercise post vs. control post) [109]
Fronto-temporal fiber tracts (exercise group: post vs. pre) [108]
Activation:
Precentral gyrus, posterior parietal cortex, precuneus (exercise post vs. control post)
Anterior cingulate cortex, dlPFC, insula (exercise post vs. control post)
[110, 112]
Network synchrony:
DMN, ECN and motor network (exercise post vs. control post)
[111]
7
8
9
10
11
12
13
14
15
16
17
18
Mean Age [years]

Supplementary Figure 2

Brain changes after diverse interventions in children and adolescents with excess weight. FA = fractional anisotropy; ECN = executive control network; DMN = default mode network; dlPFC = dorsolateral prefrontal cortex. "↑" indicates a greater metric or an increase over time in the intervention. "↓" indicates a smaller metric or a decrease over time in the intervention. " — " means no changes in metrics. "Exercise" section includes aerobic and resistance exercise. "Eating behavioral strategies" section includes 1) having breakfast instead of skipping it, 2) using food intake reduction device training, which reduces portion size and eating speed by feedback technique. "Weight-loss program intervention" section includes interventions combining exercise with dietary restriction, cognitive behavioral therapy, family management. Activation means brain response to visual food cue task, risky-gains task, antisaccade task requiring participants to view the mirror orientation of the displayed image; and flanker task, which requires participants to identify the orientation of the central symbol and press a button using the corresponding hand. Intervention responders: reduction of BMI-SDS>0.2; non-responders: reduction of BMI-SDS<0.2. Post: after intervention; pre: before intervention. Straight font means that the study used whole brain analysis. Italic font means that the study used region of interest analysis.

Supplementary Table 1
Description of studies investigating the role of parental metabolic status on pediatric obesity.

Author	Study design	Study sample	Age (M, SD)/age range	Tanner stage (M)	Weight status: BMIz/ BMI/ BMI%/ BMI cole score (M, SD)	Gender (% female)	Imaging modality	Paradigm	Fasting before fMRI	Analysis methods	Main outcomes
Thapaliya et al. 2021 [86]	C.S.	83 (25 HR, 22 LR, 36 OW/OB)	HR: 15.9 (1.2); LR: 16.5 (1.3); OW/OB: 16.0 (1.2)	1-5	BMI%: HR: 41.4 (27.5); LR: 42.7 (25.6); OW/OB: 94.5 (4.5)	49	Structural MRI			Whole-brain analysis	Lean adolescents with high risk vs. low risk and healthy weight: ↓ Gray/white matter volume or cortical thickness in the **postcentral gyrus**, **opercular cortex**, **ACC**, **precuneus**
Carnell et al. 2017 [87]	C.S.	36 (16 HR, 10 LR, 10 OW)	HR: 15.5 (1.4); LR: 16.0 (1.9); OW/OB: 15.8 (1.8)	3-5	BMI%: HR: 53 (23); LR: 51 (23); OW/OB: 95 (4)	56	fMRI	food/ nonfood words	N.S.	Whole-brain analysis	Lean adolescents with high risk vs. low risk and healthy wight: ↓ Activation in the **dlPFC**, **dACC** ↓, in response to food (vs. non-food) words
Luo et al. 2021 [88]	C.S.	76 (in total)	All: 8.62 (1.02)	1-4	BMI%: All: 69.78 (26.19)	63	fMRI	visual food cue	12-h overnight fast	ROI analysis	Maternal current BMI **positively** correlated with decreased food cue reactivity in **dlPFC** and **ACC** after glucose ingestion
Alves et al. 2020 [89]	C.S.	88 (in total)	All: 8.37 (0.89)	1-3	BMIz: All: 0.73 (1.09)	58	Structural MRI			ROI analysis	Boys, but not girls: Maternal prepregnancy BMI **positively** correlated with total **hippocampus** and **subfield** volumes

(continued on next page)

Author	Study design	Study sample	Age (M, SD)/age range	Tanner stage (M)	Weight status: BMIz/ BMI/ BMI%/ BMI cole score (M, SD)	Gender (% female)	Imaging modality	Paradigm	Fasting before fMRI	Analysis methods	Main outcomes
Lynch et al. 2021 [90]	C.S.	117 (in total)	Unexposed: 8.74 (1.11); GDM-exposed: 8.40 (0.89)	N.S.	BMIz: Unexposed: 0.72 (0.97); GDM-exposed: 0.82 (1.16)	57	Structural MRI			ROI analysis	Children exposed to GDM vs. unexposed: ↓ Radial thickness in the left **inferior body of the hippocampus**
Luo et al. 2023 [91]	C.S.	8521 (in total)	Unexposed: 9.92 (0.63); GDM-exposed: 9.92 (0.62)	1-4	N.S.	49	Structural MRI			Whole-brain analysis	Children exposed to GDM vs. unexposed: ↓ Volume in the **rostral middle frontal gyrus** and **superior temporal gyrus**
Page et al. 2019 [92]	L.S.	91 (in total)	All: 8.4 (0.9)	N.S.	BMIz: All: 0.75 (1.09)	60	ASL			ROI analysis	Children exposed to GDM before 26 weeks' gestation vs. unexposed: ↑ **Hypothalamic** blood flow in response to glucose; hypothalamic response to glucose **positively** correlated with child's BMI in 1 year
Luo et al. 2021 [94]	C.S.	159 (in total)	All: 8.50 (0.96)	1-4	BMIz: All: 0.80 (1.10)	60	fMRI	visual food cue	12-h overnight fast	ROI analysis	Children exposed to GDM: Food cue reactivity in the **OFC** **positively** correlated with energy intake
Chandrasekaran et	C.S.	122 (in total)	All: 8.8 (1.17)	1-4	BMIz: All: 0.9 (1.12)	57	Structural MRI			ROI analysis	Children exposed to GDM before 26 weeks' gestation vs. unexposed:

al. 2022 [93]	↑ gliosis in the **mediobasal hypothalamus**

Note. "↑" indicates a greater metric. "↓" indicates a smaller metric; ACC = anterior cingulate cortex; ASL = arterial spin labeling; BMI = body mass index; BMIz = BMI z-score; C.S.= cross-sectional study; DTI = diffusion tensor imaging; dlPFC = dorsolateral prefrontal cortex; dACC = dorsal ACC; fMRI = functional MRI; FC = functional connectivity; FA = fractional anisotropy; HW = healthy weight; HR = high risk; L.S.= longitudinal study; LR = low risk; M = mean; MRI = magnetic resonance imaging; MD = mean diffusivity; N.S. = not specified; OW = overweight; OB = obesity; OFC = orbital frontal cortex; rs-fMRI = resting state fMRI; ROI = region of interest; SD = standard deviation.

Supplementary Table 2 Description of studies examining effects of different non-pharmacological interventions on the brains of children and adolescents with excess weight.

Author	Study design	Study sample	Age (M, SD)/age range	Tanner stage (M)	Weight status: BMIz/ BMI/ BMI%/ BMI cole score (M, SD)	Gender (% female)	Imaging modality	Paradigm	Fasting before fMRI	Analysis methods	Main outcomes
Esteban-Cornejo et al. 2019 [95]	C.S.	Sample 1: 100; Sample 2: 242	Sample 1: 10.0 (1.1); Sample 2: 8.6 (0.5)	N.S.	BMI: Sample 1: 26.7 (3.7); Sample 2: HW: 16.2 (1.4); Sample 2 OW/OB: 22.5 (3.4)	48	Structural MRI			Whole-brain analysis	Cardiorespiratory fitness, motor fitness, and muscular fitness **positively** correlated with white matter volume in the **inferior/superior temporal gyrus**, **inferior fronto-opercular gyrus**, **insula**, **caudate**, **supramarginal gyrus**
Esteban-Cornejo et al. 2017 [96]	C.S.	101 (in total)	All: 10.0 (1.1)	N.S.	BMI: All: 26.8 (3.6)	40	Structural MRI			Whole-brain analysis	Children with OW/OB: Cardiorespiratory fitness, speed-agility **positively** correlated with grey mater volume in **frontal regions**, **temporal regions**, **hippocampus**, **caudate**
Cadenas-Sanchez et al. 2023 [97]	C.S.	110 (in total)	All: 10.0 (1.1)	N.S.	BMI: All: 26.7 (3.6)	36	Structural MRI			ROI analysis	Physical activity **positively** related to radial distance in the **putamen**, **thalamus**, **pallidum**; cardiorespiratory fitness **positively** related to radial distance in the **amygdala**; speed-agility **positively** related to radial distance in the **NAcc**

(continued on next page)

Author	Study design	Study sample	Age (M, SD)/age range	Tanner stage (M)	Weight status: BMIz/ BMI/ BMI%/ BMI cole score (M, SD)	Gender (% female)	Imaging modality	Paradigm	Fasting before fMRI	Analysis methods	Main outcomes
Esteban-Cornejo et al. 2019 [98]	C.S.	101 (in total)	All: 10.02 (1.14)	N.S.	BMI: All: 26.76 (3.65)	40	Structural MRI			Whole-brain analysis	Children with OW/OB: cardiorespiratory fitness and speed-agility **positively** related to **overall cortical thickness**
Esteban-Cornejo et al. 2021 [99]	C.S.	99 (in total)	All: 10.0 (1.1)	N.S.	BMI: All: 26.7 (3.7)	39	fMRI	Resting state		ROI/ Whole-brain analysis	Children with OW/OB: Speed-agility **positively** related to rs-FC between the **posterior hippocampus** and **precentral gyrus**, **ACC**; cardiorespiratory fitness **positively** related to rs-FC between **the anterior hippocampus** and **superior frontal gyrus**
Logan et al. 2022 [100]	C.S.	121 (in total)	All: 9.3 (1.1)	1.44 ± 0.5	BMI: All: 19.0 (4.2)	56	fMRI	Resting state		ROI analysis	Cardiorespiratory fitness **positively** related to FC between regions in the **ventral attention** and **frontoparietal networks**
Rodriguez-Ayllon et al. 2020 [101]	C.S.	104 (in total)	All: 10.04 (1.15)	N.S.	BMI: All: 26.68 (3.63)	41	DTI			Whole-brain analysis	Children with OW/OB: Physical fitness not associated with global FA; muscular fitness **positively** correlated with FA in the **lateral frontal lob**e

(continued on next page)

Author	Study design	Study sample	Age (M, SD)/age range	Tanner stage (M)	Weight status: BMIz/ BMI/ BMI %/ BMI cole score (M, SD)	Gender (% female)	Imaging modality	Paradigm	Fasting before fMRI	Analysis methods	Main outcomes
Adelantado-Renau et al. 2023 [102]	C.S.	100 (in total)	All: 10.0 (1.2)	N.S.	BMI: All: 26.6 (3.5)	41	Structural MRI			ROI analysis	Children with OW/OB: brain-derived neurotrophic factor (BDNF) was **positively** associated with hippocampal volume in fit children but negatively associated in unfit children
Haapala et al. 2024 [103]	C.S	100 (in total)	All: 10.1	N.S.	N.S.	N.S.	Structural MRI			Whole-brain analysis	Children with OW/OB: Cardiorespiratory fitness **positively** related to t**otal grey mater volume**
Rodriguez-Ayllon et al. 2020 [104]	C.S.	103 (in total)	All: 10.02 (1.15)	N.S.	BMI: All: 26.72 (3.62)	41	DTI			Whole-brain analysis	Children with OW/OB: physical activity **positively** related to **global FA**
Brooks et al. 2021 [105]	C.S.	5955 (in total)	9-10	N.S.	BMI: All: 17.37 (4.28)	51	fMRI	Resting state		Whole-brain analysis	Physical activity **positively** related to node clustering of the **DMN**, **ECN** and **SN**
Alves et al. 2021 [106]	C.S.	100 (in total)	All: 8.51 (1.00)	1-3	BMIz: All: 0.75 (1.09)	59	DTI			Whole-brain analysis	Children exposed to maternal OW/OB: Vigorous physical activity **positively** correlated with **global FA** and intelligence

(continued on next page)

Author	Study design	Study sample	Age (M, SD)/age range	Tanner stage (M)	Weight status: BMIz/ BMI/ BMI %/ BMI cole score (M, SD)	Gender (% female)	Imaging modality	Paradigm	Fasting before fMRI	Analysis methods	Main outcomes
Ortega et al. 2022 [107]	L.S.	109 (in total)	All: 10.0 (1.1)		BMI: All: 26.8 (3.6)	41	Structural MRI			ROI analysis	Children with OW/OB (post vs. pre): No effect of exercise on the volume of the **hippocampus**, the shape of **DS**, **VS**, **amygdala**; ↑ Intelligence, cognitive flexibility
Krafft et al. 2014 [108]	C.S.	18 (Exercise group: 10; Control group: 8)	Exercise group: 9.9 (0.6); Control group: 9.4 (0.8)	N.S.	BMI: Exercise group: 25.6 (3.7); Control group: 27.2 (10.4)	50	DTI			ROI analysis	Exercise group (post vs. pre): ↑ FA in **superior longitudinal fasciculus**, **positively** correlated with attendance
Schaeffer et al. 2014 [109]	C.S.	18 (Exercise group: 10; Control group: 8)	Exercise group: 9.9 (0.6); Control group: 9.4 (0.8)	N.S.	BMI: Exercise group: 25.6 (3.7); Control group: 27.2 (10.4)	50	DTI			ROI analysis	Exercise (post) vs. control group (post): ↑ FA in **uncinate fasciculus**
Krafft et al. 2014 [110]	C.S.	43 (Exercise group: 24; Control group: 19)	Exercise group: 9.7 (0.8); Control group: 9.9 (0.9)	N.S.	BMIz: Exercise group: 1.91 (0.42); Control group: 1.93 (0.57)	65	fMRI	Antisaccade + flanker task	N.S.	Whole-brain analysis	Exercise (post) vs. control group (post): ↓ Activation in the **pre/postcentral gyrus**, **posterior parietal cortex (**antisaccade task); ↑ Activation in the **ACC**, **superior frontal gyrus**, **middle prefrontal gyrus** (flanker task)

(continued on next page)

Author	Study design	Study sample	Age (M, SD)/age range	Tanner stage (M)	Weight status: BMIz/ BMI/ BMI %/ BMI cole score (M, SD)	Gender (% female)	Imaging modality	Paradigm	Fasting before fMRI	Analysis methods	Main outcomes
Krafft et al. 2014 [111]	C.S.	22 (Exercise group: 13; Control group: 9)	Exercise group: 9.5 (0.6); Control group: 9.6 (0.9)	N.S.	BMI ≥ 85th percentile	68	fMRI	Resting state		ROI analysis	Exercise (post) vs. control group (post): ↓ Network synchrony in **DMN**, **ECN**, **motor network**
Davis et al. 2011 [112]	C.S.	18 (Exercise group: 11; Control group: 9)	All: 9.6 (1.0)	N.S.	BMI: All: 25.3 (6.0)	40	fMRI	Antisaccade task	N.S.	ROI analysis	Exercise (post) vs. control group (post): ↑ Activation in the **PFC**, ↓ activation in the **posterior parietal cortex**; ↑ Executive function
Leidy et al. 2011 [114]	L.S.	10 (in total)	All: 15 (1)	N.S.	BMI%: All: 93.1 (1.4)	100	fMRI	Visual food cue	Eat 3 h prior fMRI	Whole-brain analysis	Breakfast vs. skipping: ↓ Food cue reactivity in the **amygdala**, **cingulate**, **hippocampus**, after breakfast consumption
Hinton et al. 2018 [115]	L.S.	19 (in total)	11-18	N.S.	BMI ≥ 95th percentile	63	fMRI	Visual food cue	N.S.	ROI analysis	Meal weighing device training (post) vs. standard care group (post): ↓ Food cue reactivity in the **striatum** and **temporal occipital fusiform cortex** at 60/90 min following glucose consumption

(continued on next page)

Author	Study design	Study sample	Age (M, SD)/age range	Tanner stage (M)	Weight status: BMIz/ BMI/ BMI %/ BMI cole score (M, SD)	Gender (% female)	Imaging modality	Paradigm	Fasting before fMRI	Analysis methods	Main outcomes
Augustijn et al. 2019 [116]	L.S.	43 (24 HW, 19 OB)	pre HW: 9.6 (1.2); OB: 9.4 (1.0); post HW: 10.0 (1.2); OB: 9.8 (1.0)	1-3	BMI: pre HW: 16.90 (1.17); OB: 31.50 (4.43); post HW: 16.91 (1.21); OB: 25.71 (3.73)	37	Structural MRI+DTI			Whole-brain analysis	OB group (post vs. pre): ↑ Gray matter volume of **cerebellar** and **total brain**; No change in white matter organization
Mata et al. 2016 [117]	L.S.	16 (in total)	All: pre: 13.94 (1.65)	N.S.	BMI: pre: 27.95 (4.29); post: 26.46 (4.37)	75	fMRI	Risky-Gains Task	N.S.	ROI analysis	↑ **Insula** activation during risky decision-making from baseline to post-intervention **positively** correlated with reduction in BMI and fat percentage
Kinder et al. 2014 [118]	L.S.	14 (in total)	pre: 13.15 (2.51); post: 14.48 (2.44)	N.S.	BMI: pre: 28.89 (3.80); post: 28.53 (4.28)	86	fMRI	Visual food cue	N.S.	ROI analysis	Therapeutic responder (post) vs. non-responder (post): ↑ Brain response to food cues in the **putamen**, ↑ response to sport images in the **inferior frontal gyrus, the ventral premotor cortex** Therapeutic responder (post vs. pre) ↑ I**nsula, cerebellum** activation to pleasant images

(continued on next page)

Author	Study design	Study sample	Age (M, SD)/age range	Tanner stage (M)	Weight status: BMIz/ BMI/ BMI %/ BMI cole score (M, SD)	Gender (% female)	Imaging modality	Paradigm	Fasting before fMRI	Analysis methods	Main outcomes
Schur et al. 2020 [119]	L.S.	37 (in total)	All: 10.5 (0.9)	N.S.	BMI: pre: 29.5 (7.0); post: 28.1 (7.6)	38	fMRI	Visual food cue	N.S.	ROI/ whole-brain analysis	Pre-intervention reduction in food cue reactivity to a meal in the **amygdala**, **DS**, **VS**, **insula**, **medial OFC**, **substantia nigra/VTA**, **positively** correlated with BMI declines after a 6-month intervention
Martín-Pérez et al. 2020 [120]	L.S.	70 (36 HW, 34 OW/OB)	HW: 16.50 (1.40); OW/OB: 16.44 (1.66)	N.S.	BMI%: HW: 50.33 (19.31); OW/OB: 93.74 (4.27)	54	fMRI	Resting state		ROI analysis	Pre-intervention rs-FC **between the left central amygdala nuclei and midbrain positively** correlated with weight loss after 3-month intervention

Note. "↑" indicates a greater metric or an increase over time. "↓" indicates a smaller metric or a decrease over time; ACC = anterior cingulate cortex; BMI = body mass index; BMIz = BMI z-score; C.S.= cross-sectional study; DTI = diffusion tensor imaging; DS = dorsal striatum; dlPFC = dorsolateral prefrontal cortex; DMN = default mode network; ECN = executive control network; fMRI = functional MRI; FC = functional connectivity; FA = fractional anisotropy; HW = healthy weight; L.S.= longitudinal study; M = mean; MD = mean diffusivity; MRI = magnetic resonance imaging; N.S. = not specified; NAcc = nucleus accumbens; OW = overweight; OB = obesity; OFC = orbital frontal cortex; PFC = prefrontal cortex; ROI = region of interest; RD = radial diffusivity; rs-fMRI = resting state fMRI; SD = standard deviation; SN = salience network; VS = ventral striatum; VTA = ventral tegmental area.
